# Environmentally induced stress affects fitness of bold and shy alike: A long‐term study of personality and feather corticosterone in Arctic‐breeding kittiwakes

**DOI:** 10.1111/1365-2656.70225

**Published:** 2026-02-24

**Authors:** Frederick C. Mckendrick, Samantha C. Patrick, Sébastien Descamps, Alexis P. Will, Kathryn E. Arnold, Stephanie M. Harris, Alexander S. Kitaysky

**Affiliations:** ^1^ School of Environmental Sciences University of Liverpool Liverpool UK; ^2^ Norsk Polarinstitutt, Fram Centre Tromsø Norway; ^3^ World Wildlife Fund US Arctic Program Fairbanks Alaska USA; ^4^ Department of Biology and Wildlife, Institute of Arctic Biology University of Alaska Fairbanks Fairbanks Alaska USA; ^5^ Department of Environment and Geography University of York York UK; ^6^ School of Ocean Sciences Bangor University Bangor UK; ^7^ Department of Coastal Systems NIOZ Royal Netherlands Institute for Sea Research Texel the Netherlands

**Keywords:** Atlantification, behaviour, fitness, glucocorticoids, phenotypic syndromes, Svalbard

## Abstract

1. Individual repeatable variation in behaviour, that is ‘personality’, is hypothesised to mediate how animals respond to environmental stress. However, temporal variability in local conditions and spatial constraints across the annual cycle may affect individual responses to environmental change and the subsequent impact on fitness. Here we tested how the relationships among personality, stress exposures and fitness may vary between the breeding and wintering periods in the black‐legged kittiwake (*Rissa tridactyla*), a long‐lived migratory seabird inhabiting the rapidly changing Arctic.
2. We conducted a long‐term (2013–2021) study to explore how personality, represented by boldness, was related to feather corticosterone (fCORT), an indicator of stress exposure during feather growth. To examine temporal variation in this relationship, we focused on feathers grown during the breeding, post‐breeding and pre‐breeding stages, three periods when seabirds experience varying spatial constraints. We studied the covariation in fCORT, boldness and two fitness measures (chick survival and adult return rates) while correcting for two major proxies of resource availability: the subpolar gyre and Atlantic water influx (AWI)
3. We observed a season‐dependent effect of boldness: ‘bolder’ individuals had lower fCORT concentrations than ‘shyer’ conspecifics during breeding and post‐breeding but higher levels prior to breeding. Higher fCORT during breeding occurred during years of low AWI and correlated with lower chick survival, while higher fCORT prior to breeding correlated with lower likelihood of return to the colony. Personality did not mediate inter‐annual relationships between fCORT and environmental measures or fCORT and fitness.
4. Our results highlight that while the negative effects of environmentally induced stress on fitness appeared to be ubiquitous across personality types, how and when bold and shy individuals experience stress is highly context dependent, reflecting different spatial and temporal constraints during the breeding and wintering stages.

## Graphical abstract

Quantifying how individuals differ as their environment changes around them is crucial to predict population responses to climate change. By incorporating personality, physiology and life‐history the authors show that while environmental change is likely to impact the whole population equally, when individuals are most impacted will vary across the annual cycle.

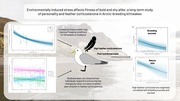

## 1 INTRODUCTION

Understanding the variability in how individuals respond to environmental stressors is necessary to quantify the effects of climate change on animal populations (Cockrem, 2022; Dingemanse & Wolf, 2013). Consistent individual variation in behaviour, commonly known as “personality”, is well documented across animal taxa (Bell et al., 2009) and is predicted to influence how sensitive individuals are to changes in their environment (Reader et al., 2007). Shyer, less aggressive and more social individuals are generally more reactive to changes around them and tend to prioritise self‐maintenance, contrasting with their proactive conspecifics that are bolder, more aggressive and prioritise reproductive output (Réale et al., 2010). Personality differences are consistent between individuals (Bell et al., 2009) and partially heritable (Dochtermann et al., 2015). Personality may influence how individuals cope with uncertainty in their surroundings via a suite of behavioural and physiological traits (Mathot et al., 2012).

Personality is hypothesised to be a key mediator of the physiological response to stressful conditions, for example allostasis (Carere et al., 2010), particularly in the face of anthropogenically induced climate change (Cockrem, 2022). The adrenocortical response is one of the first mechanisms individuals deploy in response to an environmental challenge (Ames et al., 2020) through the activation of the Hypothalamic–Pituitary–Adrenal (‘HPA’) axis and increased secretion of glucocorticoids (Wingfield et al., 1982; Wingfield & Ramenofsky, 1999). Driven by actual or perceived environmentally induced changes in metabolic demands (Jimeno & Verhulst, 2023), glucocorticoid release promotes behaviours that facilitate the maintenance of homeostasis, such as increased foraging effort and hence provides stability in physiological functioning (Angelier et al., 2007; Long & Holberton, 2004). Reactive personality types, being more responsive to their current environment, are predicted to have larger increases in glucocorticoid levels than proactive individuals (Cockrem, 2013).

To date, however, studies investigating links between personality and HPA functioning have yielded contrasting results (Arnold et al., 2016; Baugh et al., 2017; McMahon et al., 2022). This may be due in part to the inter‐annual and/or seasonal change of environmental conditions in which individuals are examined. For example, in a food‐rich environment where HPA axis activity is expected to be low (Kitaysky et al., 2010), proactive personality types—those that are bolder and more active—may have relatively higher circulating glucocorticoid levels than reactive conspecifics—which are shyer and more risk‐averse individuals—due to their increased energy expenditures (Jimeno & Verhulst, 2023). In contrast, reactive individuals may be more strongly affected by challenging environmental conditions through increased sensitivity or capacity of the HPA axis and thus have higher levels of circulating glucocorticoids than proactive individuals (Cockrem, 2013). Accounting for variation in ecological conditions may help disentangle the relationships between personality and HPA activity.

Differences in how individuals respond to environmental challenges may also differ across stages of the annual cycle, adding further complexity to the quantification of relationships between personality and HPA activity (McNamara & Houston, 2007). For instance, in migratory birds, the energetic constraints placed on individuals vary due to changes in their physical and ecological environments, such as the need to return to the breeding site when raising young (Dunn et al., 2020; Piersma, 2002). These changes in constraint may limit variation in physiological activity, leading to similar environmental responses by differing personality types (van de Pol et al., 2016). Thus, accounting for both the variation across life‐history stages and environmental quality is needed to quantify the relationships between personality and HPA activity. Differences in short‐term (minutes to hours) glucocorticoid responses provide insight into immediate responses to environmental stressors but have a limited ability to profile changes in glucocorticoid levels over time and to capture long‐term (days/weeks) changes in energetic imbalance that can influence fitness outcomes (Blas & Fairhurst, 2022). Measurements of corticosterone that are integrated over longer timescales, such as feather corticosterone (fCORT) (Bortolotti et al., 2008), may provide better insights into the effect of long‐term environmental perturbations (Blas & Fairhurst, 2022). For instance, persistent reductions in food availability increase strain on the body by continually exposing individuals to energetic imbalance, that is allostatic load, and requiring consistent release of corticosterone to activate stored energy (Johns et al., 2018). fCORT consequently integrates variation in baseline corticosterone (limitations discussed in Romero & Fairhurst, 2016) as well as stress‐induced corticosterone levels from acute stressors (Bortolotti et al., 2008) and has been shown to correlate with changes in food availability and subsequent nutritional stress (Will et al., 2014, 2018). Corticosterone is passively deposited into feathers as they grow, and thus fCORT represents the cumulative corticosterone exposure during feather growth (days to weeks depending on size of the feather) (Jenni‐Eiermann et al., 2015). fCORT both prior to and during breeding has been shown to correlate with current (López‐Jiménez et al., 2017; Monclús et al., 2020; but see Harris et al., 2017) and future reproductive performance and survival (Harms et al., 2015; Koren et al., 2011; but see Bourgeon et al., 2014). Therefore, fCORT may provide insight into how different personalities balance resource acquisition and subsequent life‐history trade‐offs. In species where moult periods are spread out across the annual cycle, fCORT also provides an opportunity to explore temporal dynamics of the relationship between personality, HPA activity and fitness.

Here, we used a long‐term study (2013–2021) of black‐legged kittiwakes, *Rissa tridactyla* (hereafter ‘kittiwake’), a long‐lived, colonial‐nesting seabird, to examine the relationship between personality and differences in fCORT across the pre‐breeding, breeding and post‐breeding moult periods. We use the trait boldness which forms one of the key components on the proactive to reactive personality spectrum with bolder birds representing more proactive individuals (Réale et al., 2007). In seabirds, including kittiwakes, boldness is shown to correlate with other traits consistent with this spectrum with bolder birds tending to exhibit less explorative movement and lower sensitivity to environmental change (Gillies et al., 2023; Harris, Descamps, Sneddon, Bertrand, et al., 2020; Kruger et al., 2019; Patrick et al., 2017). Kittiwakes experience disparate spatial constraints throughout the annual cycle, that is being central place foragers commuting between foraging and nesting sites during the breeding period, in contrast to unconstrained movements during non‐breeding periods. In seabirds, corticosterone is the primary glucocorticoid implicated in the maintenance of energy balance, and variations in fCORT reflect exposure to food limitations (Will et al., 2018; discussed further in Section 2.3 and Supporting Information S8). North Atlantic seabirds inhabit stochastic marine environments that are changing rapidly and a long‐term study is required for suitable examination of the relationships between boldness and fCORT, as daily and seasonally predictable events (i.e. temperature fluctuations, storminess) may mask relationships between HPA‐activity and broad‐scale environmental changes (Tanner & Dowd, 2019).

**FIGURE 1:**
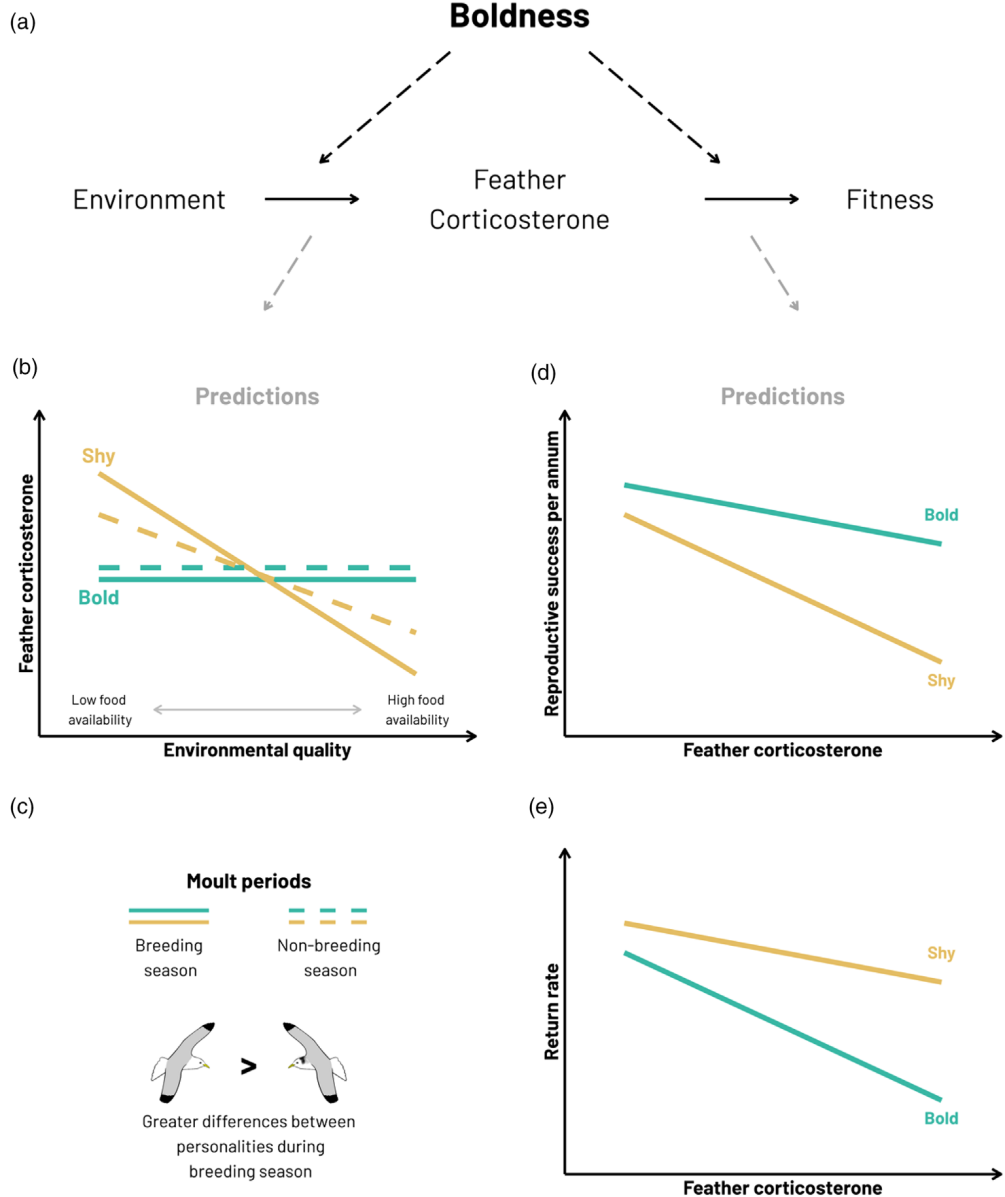
(a) Summary of the relationships investigated in this study. (b) The hypothesised relationships between proxies of resource availability, feather corticosterone (fCORT) and fitness and how these might differ across personality types. (c) The predictions for how these relationships may differ across the annual cycle. (d and e) We hypothesise negative relationships between fCORT and fitness components, as although positive and negative effects have been previously documented in experimental studies of breeding kittiwakes (Kitaysky, Kitaiskaia, et al., 2001; Schultner et al., 2013), negative effects appear to be stronger. This is a simplified framework representing our study hypotheses that focus on the possible influence of the environment, mediated through variation in fCORT, on fitness, and only portraying the responses of the extremes each boldness type. In the icons of kittiwakes in panel (c) we differentiate between breeding and non‐breeding seasons with black on the nape of the neck, as kittiwakes display in the non‐breeding season.

We hypothesise that the relationship between fCORT and boldness will depend upon both between (environmental quality) and within‐year (different life‐cycle stages) ecological variability and will have important consequences for fitness (Figure 1). Specifically, we predict that shyer, reactive individuals would show greater variation in fCORT between different quality environments compared to bolder, proactive individuals, as they are more responsive to their current environmental conditions (Cockrem, 2013; Figure 1b). The differences between the relationships of bold and shy individuals will likely be weaker in the non‐breeding periods, when individuals face fewer constraints (i.e. relieved of parental duties and no longer tied to the nest) and can therefore adopt flexible strategies to deal with environmental perturbations. We then explore whether fCORT is repeatable across the life‐cycle stages on the individual level and if the relationship between personality and fCORT varies by life‐cycle stages. We do not expect fCORT to be highly repeatable or average fCORT to differ across the bold‐shy continuum within years as we expect fCORT to be primarily driven by interannual extrinsic factors such as environmental quality.

We also investigate relationships between fCORT and fitness‐related outcomes. Previous experimental studies of kittiwakes have shown elevated corticosterone causes telomere shortening (Schultner et al., 2014) and increases the likelihood of nest failure and reduces survival (Angelier et al., 2009; Goutte et al., 2010). Therefore, we predict that higher fCORT will correlate with reductions in both reproductive performance and/or the likelihood of returning to the colony the following year (Figure 1). However, if shyer, reactive individuals are expected to prioritise their own survival over reproduction (Réale et al., 2010), we expect that shyer individuals would show a stronger negative relationship between fCORT and reproductive performance, whereas bolder, proactive individuals would show a stronger negative relationship between fCORT and return rate.

## 2 MATERIALS AND METHODS

### 2.1 Study system

**FIGURE 2:**
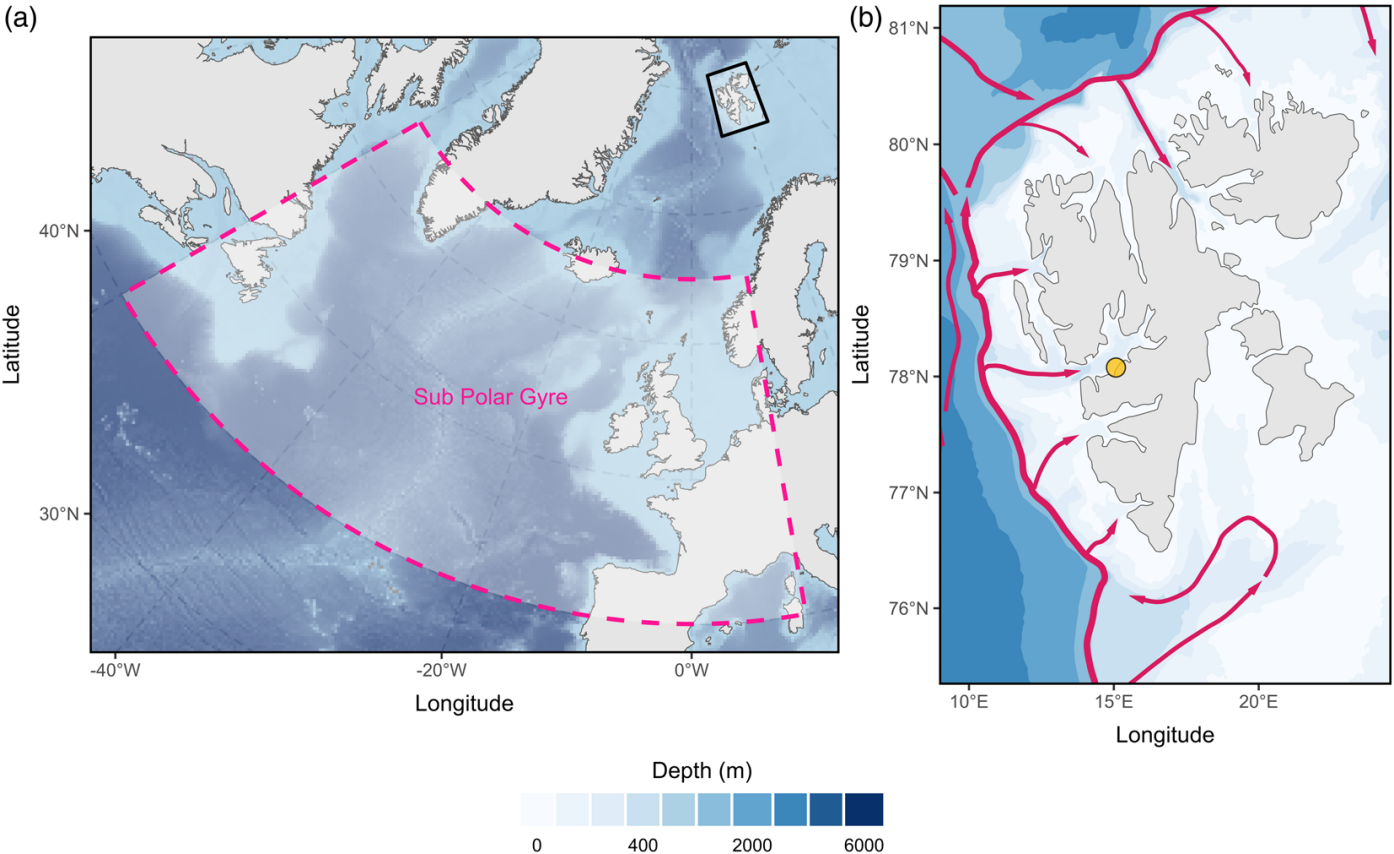
(a) The Northern Atlantic and Arctic Oceans. The pink dashed box represents the region from which sea surface height data was used to calculate the subpolar gyre index (Section 2.6.2). The black box surrounds the Svalbard Archipelago (except for Bjørnøya) where this study was conducted. (b) The Svalbard Archipelago (without Bjørnøya). The yellow dot represents the study site, Grumant. The red lines represent the West Spitsbergen current of Atlantic water. Bathymetry is represented using blue shading with darker shades indicating deeper waters.

Black‐legged kittiwakes have been monitored at Grumantbyen, hereafter Grumant, (Figure 2; 78°10′ N 15°05′ E) on the western coast of Svalbard since 2008. The colony is made up of ~20–40 breeding pairs nesting on an abandoned building. Nests are monitored twice a week from incubation in June until mid‐late chick rearing at the end of July. Individuals are sexed based on combinations of head‐bill measurements and molecular sexing using DNA extracted from blood or feather tissues (Supporting Information S1). During the non‐breeding period, kittiwakes spend most of the wintering period in the North Atlantic zone south of Greenland (Frederiksen et al., 2012). Kittiwakes are generalist seabirds primarily feeding on fish but also supplementing their diet with a variety of invertebrate prey (Vihtakari et al., 2018).

All methodology for the catching and sampling of birds at Grumantbyen was approved by the Sysselmesteren under RiS ID 361 and permitted by the Norwegian food safety authority, Mattilsynet, under permits 6293, 8616, 15,602 and 23,280.

### 2.2 Boldness

We used the trait boldness as a proxy of personality in our population with bolder individuals representing more proactive personality types (Réale et al., 2010). Boldness was quantified using a novel object test in the 2017 and 2018 breeding seasons across both incubation and chick rearing periods; a validation of the full protocol can be found in Harris, Descamps, Sneddon, Cairo, et al. (2020). In brief, this test involved a human observer presenting a blue plastic penguin (L120 mm × W90 mm × H40 mm) fixed on the end of an 8‐m long fishing pole for 60s to an individual on the nest. Individual responses to the object were recorded via video camera to identify the proportion of time spent in 5 mutually exclusive character states: (1) sitting on the nest; (2) body raised but not standing; (3) standing on the nest (legs visible); (4) off the nest but remaining on the nest ledge; (5) away from the nest (no longer visible). A single observer conducted all boldness assays, approaching the nest at ground level perpendicular to the building. Across the 2 years, a total of 80 individuals from the colony were assayed for boldness on one to seven occasions (31 individuals tested once, 15 individuals tested twice, 15 individuals three times and 19 individuals more than three times). Repeat tests occurred within and between years, with repeat tests within years occurring at least 3 days apart. Tests were primarily done during incubation and only done during chick rearing when chicks hatched between the first and latter tests.

The proportion of time spent in each behavioural state was collapsed into one variable using principal component analysis (PCA; Budaev, 2010; Harris, Descamps, Sneddon, Bertrand, et al., 2020). Principal component 1, PC1, explained 58% of the behavioural variation in the five states and was used as our measure of boldness (specific loadings can be found in Supporting Information S2). The strongest loadings represent individuals that either spent the whole assay sat down on the nest or flew away immediately following the presentation of the object (Supporting Information S2). Adjusted repeatability of boldness was calculated using linear mixed effect models that accounted for breeding stage, day of year and test number within a year and was highly repeatable for the random effect of individual ID (*R*_adj_: 0.61, 89% credible intervals: 0.49–0.72; Supporting Information S3). From this model, boldness was extracted as the mean of the posterior distribution for each individual having adjusted for the fixed effects. While the use of parameter estimates from mixed models may produce anticonservative estimates, we did not have the power to run a multivariate analysis. Repeating models using random samples from an individual posterior distribution did not change the interpretation of our findings.

### 2.3 Feather corticosterone

Individuals were caught for feather sampling during the breeding seasons between 2013 and 2021. Feather samples were taken from the first primary (hereafter ‘p1’), tenth primary (hereafter ‘p10’) and the back of the head (hereafter ‘nape’). To reduce the impact of removing feathers on flight performance and energetic expenditure, which is critical to examining fCORT–fitness relationships, only a small standardised portion of the primary feathers were clipped. Furthermore, corticosterone concentrations can vary across the length of feathers (Jenni‐Eiermann et al., 2015), and so clipping the same portion allows comparison among individuals. Specifically, a 20 mm section of p1 and p10 was cut along the proximal side of the vane, approximately 20 mm from the tip. Based on observations of kittiwakes in captivity (Kitaysky et al. unpublished), new primaries grow at a rate of ~4–5 mm/day thus a 20 mm feather fragment is likely to represent a ca. 4‐5‐day period during the breeding (p1) and post‐breeding (p10) periods. 3–5 whole nape feathers were taken between the upper nape and crown of the head. Nape feathers are typically around ~20 mm, and so all three feather samples likely represent a period of growth of similar duration.

A total of 256 feather samples (nape = 105, p1 = 80, p10 = 71) from 73 individuals were collected over the 9‐year period (individuals sampled in 1–4 years, mean = 1.4; detailed breakdown can be found in Supporting Information S4). Of the 73 feather sampled individuals, 43 had been assayed for boldness. The number of repeated feather sampling years for individuals assayed for boldness was similar to the population (individuals sampled in 1–4 years, mean = 1.7). Kittiwakes have been observed to moult p1 feathers in June–July (hereafter “Breeding”), p10 feathers during the overwintering period November–December following the breeding season (hereafter “Post‐breeding”) and nape feathers in February–March (“Pre‐breeding”), thus fCORT measures represent those three periods (Supporting Information S4; Demongin, 2016; Gonzalez‐Solis et al., 2011).

Feather samples (calamus removed from nape feathers prior to assay) were weighed to the nearest 0.001 mg (Mettler Toledo MT5 scale). On average, weights of samples were 6.075 mg (*σ* = 1.279, *n* = 80), 5.697 mg (SD = 0.698, *n* = 71) and 11.867 mg (*σ* = 5.704, *n* = 105) for p1, p10 and nape feather (2–4 per individual) samples, respectively. Feather samples were washed in isopropanol (HPLC‐grade, Sigma‐Aldrich, St. Louis, MO): 1 mL of isopropanol being added to a vial containing the feather sample, vortexed for 5 s, and then removed 50 s later. After washing, 5 mL of methanol (HPLC‐grade, Fisher Scientific, Waltham, MA) was added to each feather sample, the mixture was sonicated for 60 min at 50°C, incubated for 12 h at 50°C, followed by extraction of steroids via solid phase extraction (SPE) using BondElute C18 columns (Agilent Technologies, Santa Clara, CA). Purified extracts were then dried and reconstituted in a phosphate buffered saline with gelatine (PBSG) assay buffer (detailed analysis procedures reported elsewhere, Will et al., 2018). To account for losses of hormone during SPE, we added 2000 cpm of 3H‐labelled corticosterone (PerkinElmer NET399, Boston, MA) to each sample prior to extraction. On average recoveries were 97.43% (SD = 3.57, *n* = 256).

Feather samples were analysed in duplicates in four separate radioimmunoassays using a Sigma‐Aldrich antibody (C 8784, Saint Louis, MO, that has 100% specific binding to corticosterone and a narrow range of cross‐reactivity with other steroids, for example progesterone 12%, deoxy‐corticosterone 7%, 17‐hydroxy‐progesterone 4% and 14 other steroids 0%–2%), and incubated at 50°C for 12 h (Will et al., 2014; also see Wingfield & Farner, 1975 for a general description of the technique). Samples from each year were always processed together but distributed at random within an assay. The intra‐assay coefficient of variability (‘CV’) was 1.22% and inter‐assay CV (based on known concentration standard included in each assay) was 4.24% (Supporting Information S5). Within assays, samples were organised in batches of 48 duplicate samples, and the non‐specific and maximum binding reactions were included in each batch. Final concentrations were corrected for the percent of sample‐specific recovery, standardised to sample mass to account for differences in growth rates (ng/gram, dry weight Will et al., 2019) and normalised by log_10_ transformation prior to statistical analysis. Data transformation is regularly used and encouraged for endocrinological datasets to minimise biasing of results for cross‐sectional and longitudinal data (Miller & Plessow, 2013). Variation in raw corticosterone values is presented in Supporting Information S6.

In order to validate the corticosterone assay for kittiwake feather extracts, we confirmed that (1) changes of percent binding of serially diluted samples were parallel with those of the corticosterone standard; (2) charcoal stripping effectively removed steroids from feather extracts; and (3) serial dilution of corticosterone standard in a charcoal‐stripped feather extract did not affect the standard binding curve, that is was parallel to the standard curve (range: 7.8–2000 pg/tube) (Supporting Information S7).

In this study fCORT is interpreted as an indicator of nutritional state in kittiwakes. An important function of corticosterone in endotherms is to redirect metabolism to the utilisation of endogenous energy stores (Jimeno & Verhulst, 2023) and therefore corticosterone levels will increase when energetic intake reduces. Previous studies have found that kittiwakes exposed to reduced food consumption and low food abundance had significantly increased plasma corticosterone (Kitaysky et al., 2010; Kitaysky, Kitaiskaia, et al., 2001; Schultner et al., 2013) and lower levels of endogenous lipid resources (Kitaysky et al., 1999). In turn, circulating corticosterone was found to be passively deposited in growing feathers (Benowitz‐Fredericks et al., unpublished; Supporting Information S8), and baseline plasma corticosterone levels were positively correlated with fCORT (Will et al., in prep; Supporting Information S8). Previous studies also indicated that fCORT is an informative proxy of food availability: supplementally fed kittiwakes had lower fCORT than control individuals during food poor conditions (Kitaysky et al., unpublished; Supporting Information S8), and fCORT negatively correlated across breeding seasons with pelagic fish biomass (Will et al., 2020; Supporting Information S8). Thus, high values of fCORT most likely represent a relatively more nutritionally limited individual that is experiencing a greater energetic imbalance during feather growth.

### 2.4 Reproductive success

To quantify the relationship between fCORT and breeding output we monitored chick survival for 2 weeks post‐hatching. Reproductive success was standardised as chick survival to 15 days as it was not always possible to monitor nests to fledging (average fledgling age: 40 days). ~75% of kittiwake chick mortality occurs within the first 10 days, so our measure likely captures a large proportion of chick losses (Coulson & Porter, 1985). Individuals lay 1–3 eggs per breeding attempt (*μ* = 1.72), but no individuals in our study raised more than a single chick to 15 days. Kittiwakes typically only have one breeding attempt each season but may re‐lay if egg failure occurs early on during incubation (Gasparini et al., 2006).

### 2.5 Return rate

To measure the potential influence of fCORT on future reproductive output, we quantified the return rate to the colony the following year. Return rate could represent skipped breeding, death or permanent emigration from the colony, but adult dispersal in kittiwakes is very low and resighting rate at the colony is high, suggesting return rate may be a good estimate of survival (Vincenzi & Mangel, 2014). Resighting data show that individuals present in 1 year and not the next are twice as likely to not be seen in the colony again (244 vs. 108 occasions). Return rate was determined based on presence or absence at the colony each breeding season from resighting of colour‐ringed individuals (monitored every 3–4 days for at least 1 month each year).

### 2.6 Environmental proxies of resource availability

#### 2.6.1 Atlantic water index

The inflow of Atlantic water around the Svalbard Archipelago was quantified as the total percent contribution of Atlantic water within Isfjorden (Hop et al., 2019). Atlantic water is defined as water that is greater than 3°C with practical salinity greater than 34.65 psu (Payne & Roesler, 2019). The increasing inflow of warmer and saltier Atlantic water to the Arctic, that is greater Arctic Atlantification, increases the availability of zooplankton and therefore potential prey available to seabirds (Hop et al., 2019) and the occurrence of Atlantic fish species in the diet of kittiwakes (Vihtakari et al., 2018). Greater Atlantic water inflow is therefore predicted to benefit kittiwakes due to increased prey abundance (Descamps & Strøm, 2021) and is known to influence the foraging behaviour of seabirds breeding on Svalbard (Stempniewicz et al., 2021). Temperature and salinity measurements within the fjord were collected using vessel‐based CTD casts between July and September every year. Temperature and salinity in July and September are largely correlated with conditions earlier in the year so we are confident our measure is indicative of Atlantic water across the breeding season (Tverberg et al., 2019). The UNIS Hydrographic Database was the main source for the CTD data up to 2019 (Skogseth et al., 2020) and was complemented with data from the Norwegian Polar Institute, Institute of Oceanology Polish Academy of Sciences, Joint Norwegian‐Russian Ecosystem Survey for Barents Sea (Eriksen et al., 2018), the University Centre in Svalbard and UiT the Arctic University of Norway for 2019–2020.

An Atlantic Water Index (AWI) for a given cast *s* was calculated as follows:

(1) $${\mathrm{AWI}}_{s}=\frac{\sum 1\;m\;\text{sections with}\;\mathrm{AW}}{\sum \text{number of}\;1\;m\;\text{sections in the cast}}$$

An annual AWI for each year *y* was then calculated as

(2) $${\mathrm{AWI}}_{y}=n^{-1}\;x\;\sum_{i=1}^{n}{\mathrm{AWI}}_{s}$$

where *n* is the number of casts in a given year.

The relationship between AWI and fCORT appeared to be non‐linear, fitting a negative exponential relationship better than a linear one (Supporting Information S9). Therefore, in our models, exp(‐AWI) was also included to account for this.

#### 2.6.2 Sub‐polar gyre

As a proxy of food abundance during the non‐breeding period, we used the subpolar gyre strength. The subpolar gyre, SPG, is the predominant surface circulation feature in the Northwest Atlantic Ocean, where the majority of Svalbard kittiwakes overwinter, and has a major impact on the advection and properties of water masses within this region (Hakkinen & Rhines, 2009). Stronger SPG is correlated with increased abundance of zooplankton in the Northern Atlantic (Hátún et al., 2016), with positive effects on both the survival (Fluhr et al., 2017) and subsequent breeding success of seabirds overwintering in the North Atlantic (Hátún et al., 2017).

A SPG index (SPG‐I) was generated by Berx and Payne (2017) using maps of sea surface height data for the area between 60°W–10°E and 40–65°N (Figure 1a). Monthly mean maps were then created to represent average conditions for each month of the year. The SPG‐I was then defined as the first principal component of an empirical orthogonal function analysis applied to the sea‐level height field. Full details of how the SPG‐I was calculated can be found in (Berx & Payne, 2017). For the analysis of the influence of SPG‐I on fCORT, SPG‐I was averaged over the most likely months in which the feathers were moulted, November–December and February–March for post‐ and pre‐breeding periods, respectively.

### 2.7 Data analysis

All statistical analysis was undertaken in R (v4.2.2; R Core Team, 2024). All modelling was conducted in a Bayesian framework with the ‘brms’ package (Bürkner, 2017). All models were run across 4 chains for 10,000 iterations with a warmup of 2000 iterations and thinned every 8. All models utilise weakly informative priors (Supporting Information S10) and the use of alternative, less informative priors did not alter the interpretation of our models.

#### 2.7.1 Relationship between the environment, fCORT and boldness

We first investigated the interaction between boldness and AWI, a proxy of resource availability during the breeding season, and its relationship with fCORT. A LMM with log_10_‐transformed fCORT as the response was fitted with fixed effects of exp(‐AWI), boldness, sex, the interaction between exp(‐AWI) and boldness, as well as individual ID as a random effect.

We then investigated the interaction between boldness and the subpolar gyre index (SPG‐I), a proxy of resource availability during the non‐breeding periods (post‐ and pre‐breeding), and its relationship with fCORT. Here, two LMMs were fitted with fCORT as the response (one model for post‐breeding and one for pre‐breeding) and SPG‐I, boldness, their interaction, sex and breeding success the previous season as fixed effects and individual ID as a random effect. Breeding success in the previous season was included to account for any carry‐over effects of the increased energetic expenditure through successful breeding.

All three models were run assuming a Gaussian error distribution.

#### 2.7.2 Repeatability and within‐year variation in fCORT

To identify whether fCORT is repeatable on an individual basis and how fCORT varies across life‐stages, we fitted a LMM with log_10_‐transformed measures of fCORT from all years and moult periods as the response. We used a scaled environmental measure so that we could compare between moult periods, by mean‐centring and dividing by two standard deviations so that a low value represented either low AWI or low SPG depending on the moult period (Gelman, 2008). We used an interaction with moult period so that we were accounting for the different relationships between AWI, SPG and the respective moult periods they correspond to. To explore whether the relationship between boldness and fCORT varied by time of year, we included an interaction of boldness and moult period. There was an additional fixed effect of Sex, and both individual ID and the year the feather was moulted were included as random effects. An adjusted repeatability for fCORT was then calculated for individual ID across all feather samples, accounting for the fixed effects in the above model structure (Nakagawa & Schielzeth, 2010).

The model was run assuming a Gaussian error distribution.

#### 2.7.3 Life‐history correlates of fCORT variation

To assess the correlation between fCORT and reproductive success we fitted a LMM with chick survival as a response variable. Fixed effects included breeding fCORT, boldness, sex and the AWI within the fjord that breeding season. Only one member of a pair at a nest was caught in a single year. To assess whether boldness influences investment in reproduction, we also fit an interaction between breeding fCORT and boldness. Individual ID was included as a random effect in all three models.

To quantify potential carry‐over effects of post‐ and pre‐breeding fCORT on reproductive success the following year we again fit a GLMM with chick survival as a response variable. Fixed effects included post‐ or pre‐breeding fCORT, boldness, the interaction between fCORT and boldness, sex and the AWI within the fjord that breeding season.

The correlation between fCORT and adult return rate was quantified using three GLMMs using a binary value as the response variable. Here we used fCORT values from the breeding (year *t* − 1) and non‐breeding seasons (post‐ and pre‐breeding in year *t* − 1 and *t*, respectively) and quantified its impact on return rate in year *t* + 1. Fixed effects therefore included fCORT, boldness, their interaction, sex, reproductive success in year *t* and SPG‐I during the following overwintering period. Individual ID was included as a random effect in all three models.

All models were run assuming a Bernoulli distribution.

#### 2.7.4 Model assessment

Model convergence was assessed using several pre‐specified criteria. Firstly, we made sure model parameters exceeded a minimum effective sample size of 1000 (Bürkner, 2017). To assess convergence between and within chains we used Gelman‐rubin (Rhat <0.01) and Geweke (−2 < *Z*‐score <2) diagnostic tools, respectively (Gelman & Rubin, 1992; Geweke, 1992). We define fixed effects as statistically significant if 89% credible intervals did not overlap 0. We used 89% credible intervals as suggested in (McElreath, 2016) as they produce more reliable estimates of credible intervals, especially when effective sample sizes are lower than 10,000 (Kruschke, 2014).

## 3 RESULTS

### 3.1 Relationship between the environment, fCORT and boldness

There was no interaction between boldness and either AWI or SPG (proxies of resource availability during breeding and non‐breeding, correspondingly) on fCORT at any time of year (Breeding mean estimate: 0.31 [89% credible intervals: −0.30, 0.88]; post‐breeding: 0.23 [−0.04, 0.50], pre‐breeding: 0.10 [−0.07, 0.27]; Table 1; Supporting Information S11).

**TABLE 1:** Outputs from the models described in Section 3.1 exploring the relationship between the environment, feather corticosterone and boldness.

Response	Parameter	Estimate	5.5% CI	94.5% CI
Breeding fCORT	**Intercept** ^a^	**0.84**	**0.67**	**1.01**
	Boldness	−0.24	−0.66	0.17
	**Exp(‐AWI)**	**0.70**	**0.48**	**0.93**
	**Sex [Male]**	**−0.08**	**−0.14**	**−0.02**
	Boldness × AWI	0.30	−0.30	0.88
	***Individual ID***	***0.04***	***0.01***	***0.08***
	***Residual***	***0.13***	***0.11***	***0.15***
Post‐breeding fCORT	**Intercept** ^a^	**1.76**	**1.64**	**1.88**
	Boldness	−0.29	−0.62	0.05
	SPG‐I	−0.10	−0.20	0.00
	Sex [Male]	0.01	−0.10	0.11
	Breeding success in previous year	−0.04	−0.15	0.06
	Boldness × SPG‐I	0.23	−0.04	0.50
	***Individual ID***	***0.07***	***0.01***	***0.13***
	***Residual***	***0.18***	***0.14***	***0.22***
Pre‐breeding fCORT	**Intercept** ^a^	**1.38**	**1.30**	**1.47**
	Boldness	0.00	−0.23	0.23
	SPG‐I	0.01	−0.05	0.08
	Sex [Male]	−0.01	−0.08	0.06
	Breeding success in previous year	−0.05	−0.12	0.02
	Boldness × SPG‐I	0.10	−0.07	0.27
	*Individual ID*	*0.04*	*0.00*	*0.08*
	***Residual***	***0.16***	***0.14***	***0.19***

*Note*: Effects in bold represent statistically significant effects. Random factors and residual components are represented in italics. In all models, the intercept refers to a female individual. fCORT: Feather corticosterone, AWI: Atlantic water index, SPG‐I: subpolar gyre index. As exp(‐AWI) describes a negative exponential component, a significant positive estimate would represent a negative relationship between AWI and the response variable.

^a^ Refers to a female individual.

**FIGURE 3:**
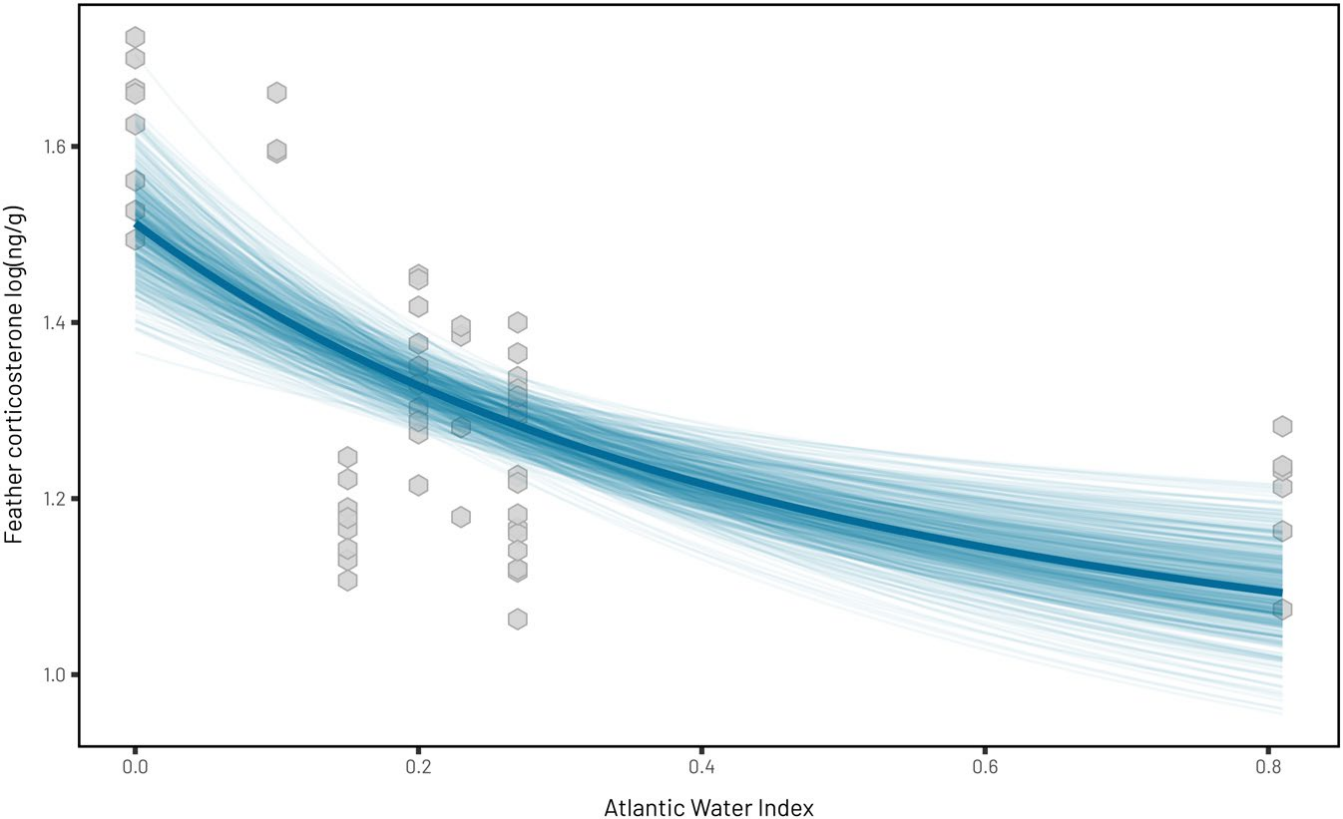
Relationship between the proportion of Atlantic water in Isfjorden and feather corticosterone during the breeding season from the first primary. Grey points represent the raw data. Thin individual blue lines represent 500 predicted relationships between AWI and corticosterone calculated with the thick line representing the mean relationship. High values of Atlantic Water Index correlate with higher zooplankton availability in Svalbard (Hop et al., 2019) and therefore likely more food available to breeding black‐legged kittiwakes.

A proxy of resource availability during the breeding season (AWI) had a significant effect on fCORT during the breeding (0.70 [0.48, 0.93]) but not the non‐breeding periods (post‐breeding: −0.10 [−0.20, 0.00]; pre‐breeding: 0.01 [−0.05, 0.08]; Figure 3; Supporting Information S12). Increasing AWI led to a reduction in fCORT during the breeding period. This relationship was not linear, showing greater decreases in fCORT at low levels of AWI before levelling off (Figure 3). fCORT differed significantly between the sexes, but only during the breeding season, with males showing lower levels of fCORT (−0.08 [−0.14, −0.02]) than females (Table 1; post‐breeding: 0.01 [−0.10, 0.11]; pre‐breeding: −0.01 [−0.08, 0.06]).

### 3.2 Repeatability and within‐year variation in fCORT

**FIGURE 4:**
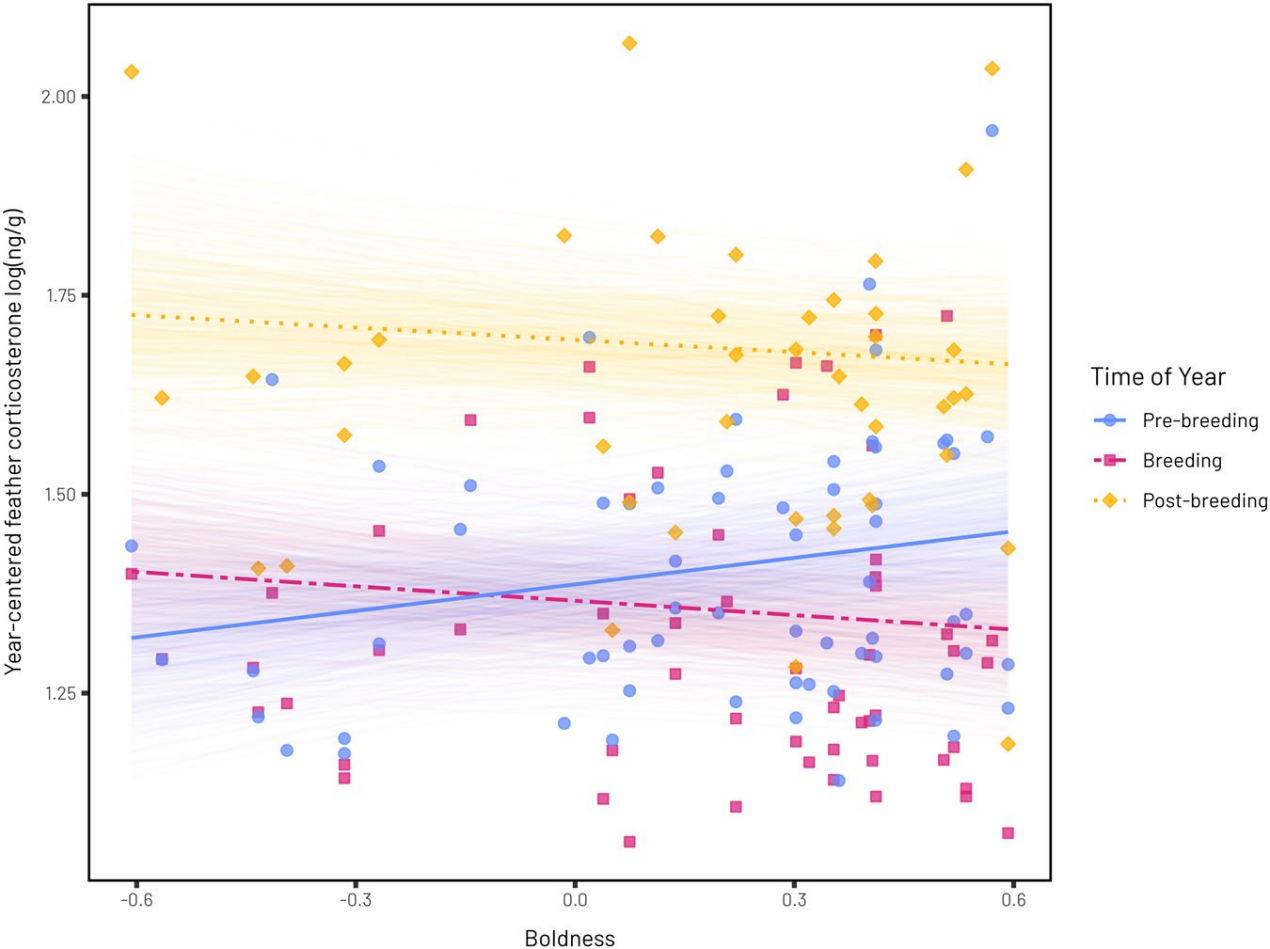
The relationship between boldness and feather corticosterone (fCORT) across different moult periods. For each moult period, thin lines represent 500 predicted relationships between boldness and fCORT calculated with the thick line representing the mean relationship. Lines are coloured and segmented by the time of year fCORT was measured (solid blue = pre‐breeding, dashed pink = breeding, dotted yellow = post‐breeding). The slopes of the relationships are statistically significant regardless of the line type (solid vs. dashed), see Section 3.2 for details.

Intra‐individual repeatability of fCORT was very low (0.08 [0.00, 0.14]). There was a statistically significant interaction between boldness and the time of year on fCORT. Whilst we observe a positive relationship between boldness and fCORT during the pre‐breeding season (0.11, [0.02, 0.20]) we find the opposite relationship in both the breeding (−0.18, [−0.30, −0.06]) and post‐breeding periods (−0.16, [−0.29, −0.04]; Table 2. Figure 4).

**TABLE 2:** Outputs from the model described in Section 2.7.2 exploring the repeatability and within‐year variation in feather corticosterone (fCORT).

Response	Parameter	Estimate	5.5% CI	94.5% CI
fCORT	Intercept^a^	1.40	*1.33*	*1.48*
	Breeding moult period	‐0.02	−0.07	0.02
	**Post‐breeding moult period**	**0.31**	**0.25**	**0.36**
	**Boldness**	**0.11**	**0.02**	**0.20**
	Sex [Male]	−0.03	−0.07	0.01
	Pre‐breeding moult period × SPG	0.06	−0.02	0.14
	**Breeding moult period** × **AWI**	**−0.10**	**−0.18**	**−0.01**
	Post‐breeding moult period × SPG	0.08	−0.04	0.19
	**Boldness** × **Breeding period**	**−0.18**	**−0.30**	**−0.06**
	**Boldness** × **Post‐breeding period**	**−0.16**	**−0.29**	**−0.04**
	***Individual ID***	***0.05***	***0.02***	***0.07***
	***Year of moult***	***0.12***	***0.08***	***0.18***
	***Residual***	***0.13***	***0.11***	***0.14***

*Note*: Effects in bold represent statistically significant effects. Random factors and residual components are represented in italics. In all models, the intercept refers to a female individual during the pre‐breeding period.

^a^ Refers to a female individual during the pre‐breeding period.

### 3.3 Life‐history correlates of fCORT variation

#### 3.3.1 Relationship between fCORT and reproductive success

**FIGURE 5:**
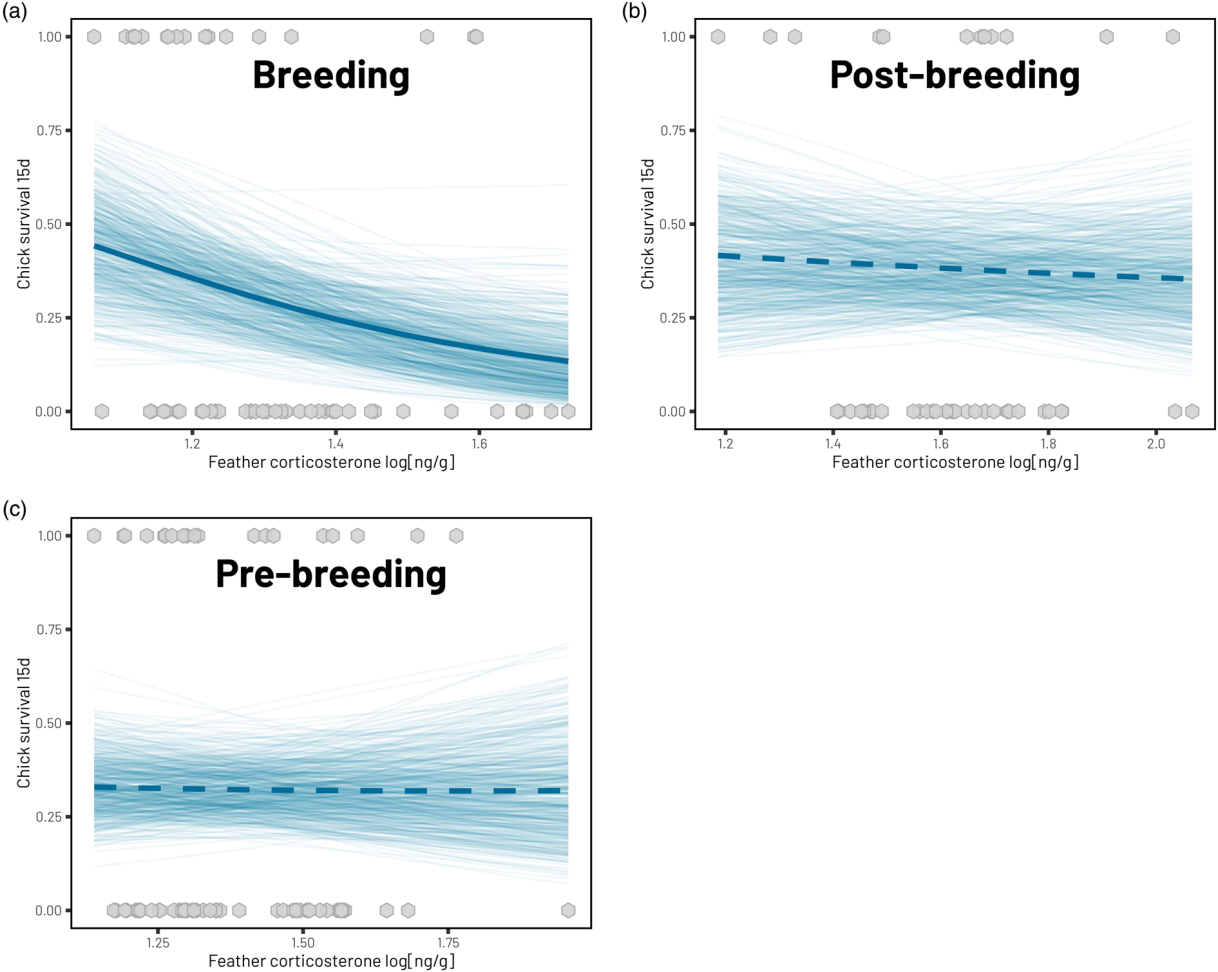
The relationship between feather corticosterone levels at three stages of the annual cycle and chick survival in the current or following breeding season (a: Breeding season, b: Post‐breeding, c: Pre‐breeding). Thick lines represent the mean predicted relationship extracted from each model, with thinner lines representing 500 predicted draws of the most likely relationship from the posterior distributions. Statistically significant relationships are denoted by solid lines (Breeding) with non‐significant results represented using dashed lines (Pre‐ and Post‐breeding). Points represent the raw data.

Boldness did not appear as a significant predictor of reproductive success in any of the models (Table 3). In the breeding season, there was a significant negative relationship between fCORT and chick survival (−3.06 [−5.47, −0.61]) but not at any other period of the year (post‐Breeding: −0.26 [−1.68, 1.18]; pre‐Breeding: −0.18 [−1.58, 1.22]; Table 3) (Figure 5). Individuals with greater levels of fCORT during the breeding season had lower reproductive success, whereby every 0.1 increase in fCORT led to a 6.4% decrease in chick survival (Figure 5).

**TABLE 3:** Outputs from the models described in Section 3.3 exploring the correlates between feather corticosterone and fitness.

Response	Parameter	Estimate	5.5% CI	94.5% CI
Chick survival to 15 days	Intercept^a^	1.76	−1.64	5.21
	**Breeding fCORT**	**−3.06**	**−5.47**	**−0.61**
	Boldness	0.71	−1.94	3.30
	Sex [Male]	−0.10	−1.26	1.08
	exp(‐AWI)	1.72	−0.99	4.35
	Breeding fCORT × Boldness	−0.58	−2.70	1.52
	***Individual ID***	***1.09***	***0.15***	***2.34***
Chick survival to 15 days	Intercept^a^	−0.37	−3.06	2.24
	Boldness	0.23	−1.24	1.69
	Post‐breeding fCORT	−0.26	−1.68	1.18
	Sex [Male]	−0.10	−1.14	0.92
	exp(‐AWI)	0.43	−1.02	1.89
	Post‐breeding fCORT × Boldness	0.04	−1.07	1.14
	***Individual ID***	***1.05***	***0.15***	***2.33***
Chick survival to 15 days	Intercept^a^	−0.76	−3.03	1.54
	Boldness	0.34	−1.02	1.73
	Pre‐breeding fCORT	−0.18	−1.58	1.22
	Sex [Male]	−0.56	−1.47	0.33
	exp(‐AWI)	0.66	−0.78	2.07
	Pre‐breeding fCORT × Boldness	0.15	−0.98	1.28
	***Individual ID***	***0.71***	***0.09***	***1.51***
Return rate year *t* + 2	Intercept^a^	4.65	−4.28	13.59
	Boldness	−0.53	−6.66	5.56
	Breeding fCORT	−1.62	−7.64	4.30
	**Sex [Male]**	**−2.49**	**−4.76**	**−0.55**
	**Breeding Success**	**2.31**	**0.50**	**4.48**
	SPG‐I	−1.47	−4.58	1.52
	Breeding fCORT × Boldness	1.45	−3.49	6.24
	***Individual ID***	1.30	0.22	2.81
Return rate year *t* + 1	Intercept^a^	0.24	−7.17	7.26
	Boldness	0.31	−6.12	6.68
	Post‐breeding fCORT	2.05	−1.75	6.15
	**Sex [Male]**	**−2.23**	**−4.21**	**−0.46**
	Breeding success	0.44	−1.29	2.32
	SPG‐I	−1.55	−3.79	0.43
	Post‐breeding fCORT × Boldness	0.25	−3.71	4.23
	***Individual ID***	***1.46***	***0.24***	***3.41***
Return rate year *t* + 1	**Intercept** ^a^	**8.20**	**3.21**	**13.49**
	Boldness	0.32	−5.54	6.40
	**Pre‐breeding fCORT**	**−3.85**	**−7.42**	**−0.52**
	**Sex [Male]**	**−1.25**	**−2.57**	**−0.02**
	Breeding success	0.73	−0.62	2.10
	SPG‐I	−1.62	−3.28	0.01
	Pre‐breeding fCORT × Boldness	0.67	−3.62	4.95
	***Individual ID***	***0.81***	***0.10***	***1.77***

*Note*: Effects in bold represent statistically significant effects. Random factors and residual components are represented in italics. All parameter estimates are represented on the logit scale. In all models, the intercept refers to a female individual. fCORT: Feather corticosterone, AWI: Atlantic water index, SPG‐I: subpolar gyre. As exp(‐AWI) describes a negative exponential component, a significant positive estimate would represent a negative relationship that plateaus between AWI and the response variable. For each response variable there are three models, one for each measure of fCORT at different periods throughout the year (Breeding, Post‐breeding and Pre‐breeding).

^a^ Refers to a female individual.

#### 3.3.2 Relationship between fCORT and return rate to the colony

**FIGURE 6:**
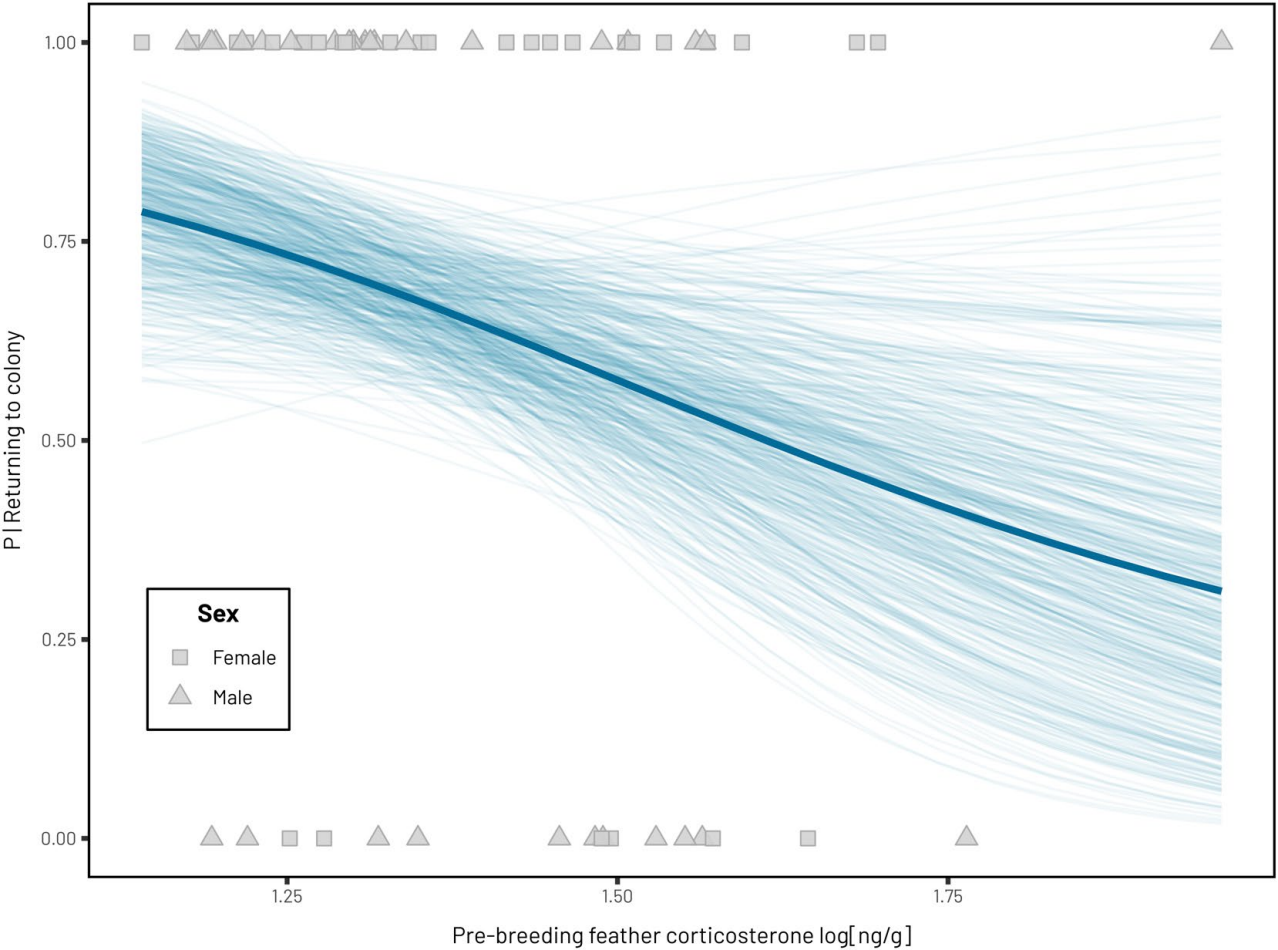
The relationship between feather corticosterone levels during the pre‐breeding period and the probability of returning to the colony the following year. Thick lines represent the mean predicted relationship extracted from each model, with thinner lines representing 500 predicted draws of the most likely relationship from the posterior distributions. All corticosterone values are represented on the log‐10 transformed scale and points represent the raw data.

Boldness did not appear as a significant predictor of return rates in any of the models (Table 3). There was a significant relationship between return rate to the colony the following year and fCORT during the pre‐breeding period (−3.85 [−7.42, −0.5]) but not fCORT during the previous post‐breeding (2.05 [−1.75, 6.15]) or breeding period (−1.62 [−7.63, 4.30]; Table 3, Figure 6). Individuals with greater levels of fCORT during the pre‐breeding period were less likely to return to the colony the following year (Figure 6), with every 0.1 increase in fCORT leading to a 1.4% decrease in return rate. In addition, within our sample, males generally showed lower return rates to the colony the following season (−2.23 [−4.21, −0.46]). When accounting for fCORT during the breeding season, individuals who bred successfully were also more likely to return to the colony the following year (2.31 [0.50, 4.48]).

## 4 DISCUSSION

Determining what drives phenotypic responses to environmental change is important to understand how individual variation is maintained within populations. Contrary to predictions, here we find individual variation in boldness does not correlate with differences in fCORT, a proxy of nutritional state, across abiotic proxies of resource availability. Independent of boldness, our abiotic proxy of resource availability correlated with fCORT during the breeding season but not any other time of year, with years of high AWI having on average lower fCORT. Lower fCORT during the breeding season was also correlated with higher reproductive success in the same year. In the pre‐breeding period, lower fCORT correlated with an increased likelihood of returning to the colony the following year. If a bird therefore had lower fCORT before the breeding season in year *t* it was more likely to return to the colony to breed in year *t* + 1. When controlling for interannual variation in fCORT, bolder birds had lower fCORT during the breeding and post‐breeding periods, a relationship that was reversed during pre‐breeding.

Corticosterone, due to its metabolic role (reviewed in Jimeno & Verhulst, 2023), is commonly suggested to be a useful indicator of nutritional state and potential allostatic imbalance in avian species, including seabirds (Blas & Fairhurst, 2022; Will et al., 2019), and kittiwakes in particular (Schultner et al., 2013). The few studies that have utilised fCORT across large spatial or temporal scales show that it is a good biomarker of resource acquisition in relation to broad‐scale environmental change (Mikkelsen et al., 2022; Treen et al., 2015). Our results add to this evidence finding that across almost a decade, fCORT is negatively related to a proxy of resource abundance during the breeding season, the AWI. fCORT is predicted to increase under food‐limiting conditions as individuals are required to use more endogenous energy stores and higher corticosterone levels can facilitate upregulation of foraging behaviour (natural corticosterone variation: Angelier et al., 2007; experimentally increased corticosterone: Kitaysky, Wingfield, et al., 2001). While kittiwakes may also experience reductions in the energetic costs of behavioural activities under the warmer sea and air temperatures that come with increased AWI (Fort et al., 2009; Skogseth et al., 2020), we expect this variation to be far smaller than what is dictated by food availability. Lower fCORT in kittiwakes under high AWI suggests that whilst potentially detrimental to Arctic‐dependent seabirds (Descamps et al., 2022), Atlantification of Svalbard may benefit wide‐spread breeding species, such as kittiwakes, with favourable foraging opportunities. While Atlantification may negatively impact the profitability of Arctic foraging hotspots such as glacier fronts (Bertrand et al., 2021), it appears that potential concurrent increases in zooplankton abundance may counteract this. Subsequently we do find that individuals with low fCORT, correlated with years with high AWI, had increased reproductive success (Figure 5). The increasing amount of Atlantic water around Svalbard over the last 20 years (Strzelewicz et al., 2022) and its subsequent correlation with low fCORT may therefore explain the relative stability of kittiwake populations in the region (Descamps & Ramírez, 2021).

The strength of the SPG is found to benefit Faroe Islands breeding kittiwakes (Hátún et al., 2017), but we found no relationship with fCORT either during the post‐ or pre‐breeding periods for kittiwakes breeding on Svalbard. Winter SPG strength may therefore not dictate food limitation and subsequent allostatic balance for Svalbard kittiwakes. Svalbard‐breeding kittiwakes exhibit much larger spatial distributions than Faroese birds (Frederiksen et al., 2012), and the spatial extent of the SPG is known to vary year to year (Biri & Klein, 2019). Spatial mismatch between the front of the SPG and the moult location of individuals may in part explain this lack of relationship. Accounting for individual variability in overwintering location is therefore the first step needed to understand what may drive fCORT variation during the non‐breeding period in Svalbard kittiwakes.

fCORT levels during the pre‐breeding period were negatively correlated with return rate in subsequent seasons, a proxy of breeding probability and likely survival (Figure 6). Individuals with high fCORT are likely to be in a worse physiological state (Johns et al., 2018). While individuals with high pre‐breeding fCORT may buffer this effect and still be able to breed (we found no statistical relationship between the two), increased energetic demands during the breeding season may sustain homeostatic imbalance within individuals arriving in poor condition (Blas & Fairhurst, 2022). Falling in line with the allostasis model of energetic requirements (McEwen & Wingfield, 2003), high fCORT, reflecting increased corticosterone exposure, represents an increased likelihood of entering allostatic overload and a reduced ability to cope with stressors (Harms et al., 2015), which could subsequently affect kittiwakes' survival.

This study provides the first example of how fCORT varies in black‐legged kittiwakes across the annual cycle, highlighting a peak in fCORT during the post‐breeding period (Supporting Information S13). Limited influence of boldness and a lack of repeatability in fCORT suggest this variation is likely driven by extrinsic factors. Energetic requirements for other Arctic seabirds (Brünnich's guillemot, *Uria lomvia*, and little auk, *Alle alle*) peak during the post‐breeding period, primarily driven by low air temperatures and increases in wind speed (Fort et al., 2009). As kittiwakes occupy similar locations and their energetic costs are affected by wind similarly to other seabirds (Elliott et al., 2014), increased corticosterone may be required for the reliance on endogenous energy stores during winter storms that impact foraging (Wingfield & Kitaysky, 2002). Given we can only sample individuals that have returned to the colony and we do not observe any carry‐over effects of post‐breeding fCORT, individuals appear to cope with the range of elevated levels of fCORT we observed and have not yet reached a critical limit that would affect their survival. Understanding how individuals may alter foraging decisions following these periods of high fCORT may provide a clue to how individuals cope with sustained periods of energetic imbalance.

Our findings show that genetic sex of individuals may play a role in both variation in fCORT and how this may impact life‐history decisions. Firstly, we show that fCORT tends to be higher in females than in males during the breeding season (Table 1). Although kittiwakes exhibit biparental care and have similar behavioural roles during breeding, egg production might be costly (Mohring et al., 2023), females are smaller and have higher energy expenditures than males during chick rearing (Welcker et al., 2015). Thus, higher levels of fCORT may be indicative of the additional energy required by females during the breeding period. Despite this, within our sample of individuals, males tended to have lower survival rates across the non‐breeding period, and this was consistent across the three models (Table 3). Sex‐biased survival is relatively common in seabird species but is highly site specific (Deakin et al., 2019; Gownaris & Boersma, 2019; Kim et al., 2011). While male kittiwakes exposed to high corticosterone experienced larger loss of telomeres compared to females (Schultner et al., 2014), and shorter telomeres have recently been found to associate with lower return rate of male kittiwakes (Benowitz‐Fredericks et al., 2022), a better understanding of the lower male return rates and its potential implications within our study population requires further investigation.

We did not find any evidence that boldness influenced interannual variation in fCORT; however, boldness was related to variation in fCORT at different times throughout the annual cycle. Shyer individuals showed greater fCORT when more spatially constrained both during the breeding season, when they are tied to the nest, and during the post‐breeding period, when they are moulting feathers important for flight (Gonzalez‐Solis et al., 2011). Shyer individuals may therefore be less effective competitors for food resources or show different foraging strategies compared to their bolder counterparts when unable to move freely within the landscape (Kurvers et al., 2009). While increased corticosterone may reflect increased investment in reproduction, baseline and stress‐induced plasma corticosterone levels peak at different reproductive stages in different years in kittiwakes, suggesting they reflect changing environmental conditions rather than consistent costs of reproduction (Kitaysky et al., 2010; Lanctot et al., 2003). We cannot rule out any effects of spatial segregation by boldness types that may influence accessibility of food during this period (Schirmer et al., 2020), but we observe that when individuals are less spatially constrained during the pre‐breeding period, shyer individuals have lower fCORT. Quantifying the influence of boldness on the space use of kittiwakes during the non‐breeding season will potentially provide a mechanistic link between boldness and fCORT.

To date, this is one of, if not the first study to investigate the relationship between fCORT and any personality trait. We find limited evidence for the hypothesised relationship between the two, at least for the focal personality trait, boldness. How any personality trait integrates with HPA activity is not well known. Previous studies show positive, negative or no covariation between personality and baseline and/or stress‐induced corticosterone (Arnold et al., 2016; Baugh et al., 2017; Carere et al., 2010; Grace & Anderson, 2014). Covariations between any measure of personality and HPA activity are likely to be highly species specific given that repeatability in corticosterone levels varies significantly between study species and the type of measurement, either baseline, stress‐induced or long‐term (reviewed in Taff et al., 2018). While Taff et al. (2018) show that long‐term measures of corticosterone are generally more repeatable (0.44 compared to 0.38 and 0.18 for stress‐induced and baseline, respectively), no long‐term studies measuring fCORT have a higher repeatability than 0.1, highlighting how fCORT will largely reflect external factors and any correlation with behavioural traits such as personality is likely to be highly context dependent. Glucocorticoid secretion is also not the only response or hormone involved in stress responses, and personality may be more related to physiological proxies linked to instant reactivity such as catecholamines (Kelly & Goodson, 2015). Future studies in kittiwakes should explore the relationship between catecholamines, glucocorticoids and personality using experimental set‐ups in order to tease apart the drivers of physiological stress between individuals (e.g. in Schultner et al., 2013). An improved framework for interpreting the relationship between personality and fCORT (or other long‐term indicators of glucocorticoid release) must also account for the spatial distribution of individuals and timing of moult, especially during periods where individuals can move freely throughout their environment with limited constraints. Given high fCORT can be a key indicator of fitness (López‐Jiménez et al., 2017; Monclús et al., 2020; Romero & Fairhurst, 2016; this study), it may provide a mechanistic link for how personality may influence the trade‐off between reproductive success and survival.

## AUTHOR CONTRIBUTIONS

Frederick C. Mckendrick, Samantha C. Patrick, Sébastien Descamps, Stephanie M. Harris, Alexander S. Kitaysky, Alexis P. Will and Kathryn E. Arnold developed the ideas present in the manuscript. Stephanie M. Harris conducted boldness assays. Sébastien Descamps coordinated field operations and managed access to the Grumantbyen kittiwake colony. Alexander S. Kitaysky and Alexis P. Will were responsible for all the lab work undertaken. Frederick C. Mckendrick developed and conducted all statistical analyses undertaken. Frederick C. Mckendrick wrote the first draft of the paper and took the lead on writing. All authors contributed critically to the drafts and gave final approval for publication.

## CONFLICT OF INTEREST STATEMENT

All authors have no conflicts of interest that may influence the findings of this research.

## STATEMENT ON INCLUSION

Our study was made possible through Norwegian collaboration, Svalbard being part of the Kingdom of Norway. All fieldwork on Svalbard was facilitated through collaboration with the Longyearbyen branch of the Norsk Polarinstitutt. All work was approved by local governance on Svalbard, namely the office of the Sysselmesteren.

## Supporting information


**Supporting Information S1.** Sex determination of black‐legged kittiwakes.
**Supporting Information S2.** Boldness principal component analysis.
**Supporting Information S3.** Repeatability of boldness.
**Supporting Information S4.** Breakdown of feathers sampled in given years.
**Supporting Information S5.** Inter‐ and intra assay coefficient of variation between corticosterone measures.
**Supporting Information S6.** Raw corticosterone values.
**Supporting Information S7.** Validation of the radioimmunoassay for kittiwake feather corticosterone.
**Supporting Information S8.** Feather corticosterone as an indicator of nutritional stress.
**Supporting Information S9.** Linear versus negative exponential relationship between AWI and feather corticosterone.
**Supporting Information S10.** Model priors.
**Supporting Information S11.** Relationship between boldness and feather corticosterone levels across the annual cycle.
**Supporting Information S12.** Environmental determinants of corticosterone variation.
**Supporting Information S13.** Variation in corticosterone across the annual cycle.

## Data Availability

All data and code used in this manuscript are available on figshare here: https://doi.org/10.6084/m9.figshare.31047331 (Mckendrick et al., 2026). The repository for the subpolar gyre dataset can be found here: https://doi.org/10.7489/1806‐1.
