# Supplementary material for: Environmentally induced stress affects fitness of bold and shy alike: A long‐term study of personality and feather corticosterone in Arctic‐breeding kittiwakes
Source: J Anim Ecol. 2026 Feb 24;95(5):763–81. doi: 10.1111/1365-2656.70225 (PMC13145314; doi:10.1111/1365-2656.70225)
Supplement: Supplementary file 1 — Supporting Information S1. Sex determination of black‐legged kittiwakes. Supporting Information S2. Boldness principal component analysis. Supporting Information S3. Repeatability of boldness. Supporting Information S4. Breakdown of feathers sampled in given years. Supporting Information S5. Inter‐ and intra assay coefficient of variation between corticosterone measures. Supporting Information S6. Raw corticosterone values. Supporting Information S7. Validation of the radioimmunoassay for kittiwake feather corticosterone. Supporting Information S8. Feather corticosterone as an indicator of nutritional stress. Supporting Information S9. Linear versus negative exponential relationship between AWI and feather corticosterone. Supporting Information S10. Model priors. Supporting Information S11. Relationship between boldness and feather corticosterone levels across the annual cycle. Supporting Information S12. Environmental determinants of corticosterone variation. Supporting Information S13. Variation in corticosterone across the annual cycle. [file JANE-95-763-s001.docx]

**Environmentally induced stress affects fitness of bold and shy alike: a long-term study of personality and feather corticosterone in Arctic-breeding kittiwakes**

**Contents**

[Supplementary information S1 2](#_Toc193897623)

[Supplementary information S2 3](#_Toc193897624)

[Supplementary information S3 5](#_Toc193897625)

[Supplementary information S4 8](#_Toc193897626)

[Supplementary information S5 10](#_Toc193897627)

[Supplementary information S6 11](#_Toc193897628)

[Supplementary information S7 12](#_Toc193897629)

[Supplementary information S8 13](#_Toc193897630)

[Supplementary information S9 18](#_Toc193897631)

[Supplementary information S10 19](#_Toc193897632)

[Supplementary information S11 21](#_Toc193897633)

[Supplementary information S12 22](#_Toc193897634)

[Supplementary information S13 24](#_Toc193897635)

# Supplementary information S1

**Sex determination of black-legged kittiwakes**

All kittiwakes included in this study were molecularly sexed using either blood or feather samples collected during processing. The primers M5 (Bantock *et al*., 2008) and P8 (Griffiths *et al*., 1998) were used to determine sex from DNA extracted from tissue samples that had been amplified via polymerase chain reaction. Molecular sexing was corroborated with the use of head-bill measurements. Sexing by head-bill methods has shown to be roughly 94% accurate in sex-determination of kittiwakes (Coulson, 2009). The sex-determination thresholds from Coulson (2009) were adapted by McCully *et al*., 2023 to account for kittiwakes in Svalbard being larger than in other parts of their range. This adaptation used the mean head-bill measurement (92.1mm) as a cut-off with individuals larger being determined as males and those below being females. Individuals within 1mm of this threshold were predicted to be determined with 92% accuracy and those outside with 99% accuracy (Table S1). Full details of the adaptation can be found in McCully *et al*., (2023).

**Table S1.1.** Head-bill measurement thresholds used to corroborate molecular sexing of kittiwakes. Thresholds used to determine sex at 92% and 99% accuracy within and outside 1mm from the mean respectively.

| Threshold (mm) | Predicted Sex (% confidence) |
| --- | --- |
| < 91 | Female (99) |
| 91 - 92 | Female (92) |
| 92.1 - 93 | Male (92) |
| > 93 | Male (99) |

# Supplementary information S2

*
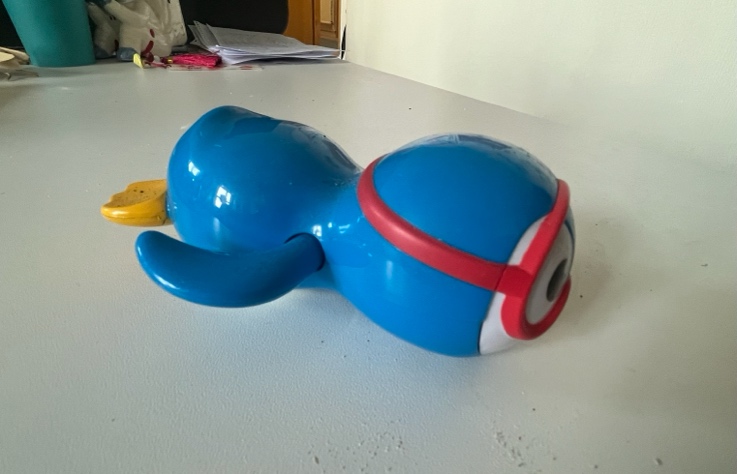

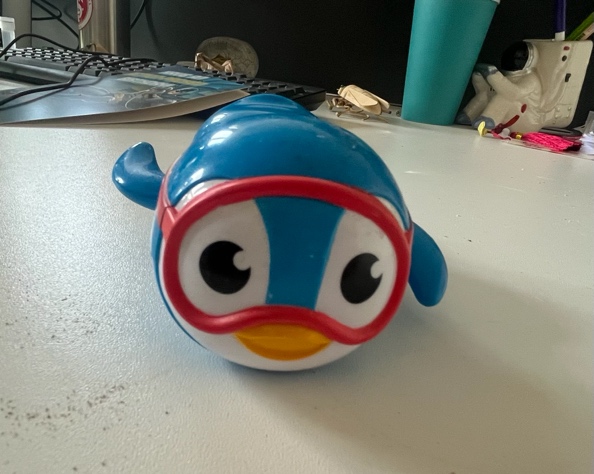
***Boldness principal component analysis**

**Figure S2.1** The novel object used in the boldness assay. The novel object measures L120mm x W90mm x H40mm. Can be found here: <https://www.munchkin.co.uk/products/swimming-scuba-buddy>.

In order to create a single measure of behaviour from the novel object test we collapsed our observed 5 behavioural states using principal component analysis. A PCA was conducted using the prcomp() function in R and found principal component 1 explained 58% of the variation in behaviour (Table S2.1).

**Table S2.1** Output from a principal component analysis of the 5 behavioural states analysed using a novel object test.

|  | Principal component | | | | |
| --- | --- | --- | --- | --- | --- |
|  | 1 | 2 | 3 | 4 | 5 |
| Standard deviation | 0.5532 | 0.3737 | 0.2426 | 0.1407 | 0.0005 |
| Proportion of variance explained | 0.5837 | 0.2663 | 0.1123 | 0.0378 | 0.0000 |
| Cumulative proportion | 0.5837 | 0.8499 | 0.9622 | 1.0000 | 1.0000 |

Table S2.2 Loadings on principal component one for the five behavioural states

| Behavioural state | Loading on PC1 |
| --- | --- |
| Sitting on the nest | -0.774 |
| Body raised | 0.058 |
| Standing on the nest | 0.586 |
| Standing off nest but visible | 0.030 |
| Away from the nest | 0.627 |

# Supplementary information S3

**Repeatability of boldness**

Adjusted repeatability, the variation in behaviour explained by individual differences after controlling for fixed effects, was calculated using PC1 of the behavioural states in a linear mixed-effects model (LMM; Nakagawa and Schielzeth, 2010). Fixed effects of breeding stage (incubation vs chick-reearing), day of year and test number within a year were included alongside individual ID as a random effect (Model outputs: Table S3.1). Adjusted repeatability, R_adj_, was calculated following (Nakagawa and Schielzeth, 2010) using:

Formula 1:

$$R_{adj}= \frac{\sigma_{ID}^{2}}{\sigma_{ID}^{2}+ \sigma_{\varepsilon}^{2}}$$

Where $\sigma_{ID}^{2}$ is the between-individual variance and $\sigma_{\varepsilon}^{2}$ is the residual variance. Our estimate for R_adj_ was 0.61, 89% credible intervals: 0.49-0.72, the same as calculated by (Harris *et al*., 2020) using a frequentist method.

Posterior estimates of individual boldness were then extracted from the individual ID random effect so we could have values of both mean boldness and the uncertainty surrounding this (Figure S3.1).

The repeatability model assumed a Gaussian distribution and were ran using weakly informative priors for both the fixed and random effects (Intercept: Normal[µ=0, σ =1], fixed effects: Normal[0,0.5], random effects: half-cauchy[0.5,0.5], residual: half-cauchy[0.5,0.5]).

**Table S3.1** Outputs from boldness repeatability model.

| **Term** | **Estimate** | **89% Credible interval** |
| --- | --- | --- |
| Intercept | -0.02 | -0.22, 0.19 |
| Stage [incubation] | 0.09 | -0.09, 0.27 |
| Test number | 0.01 | -0.04, 0.06 |
| Julian day | 0.00 | -0.17, 0.16 |
| Individual ID | 0.45 | 0.35, 0.56 |
| Residual | 0.36 | 0.31, 0.41 |


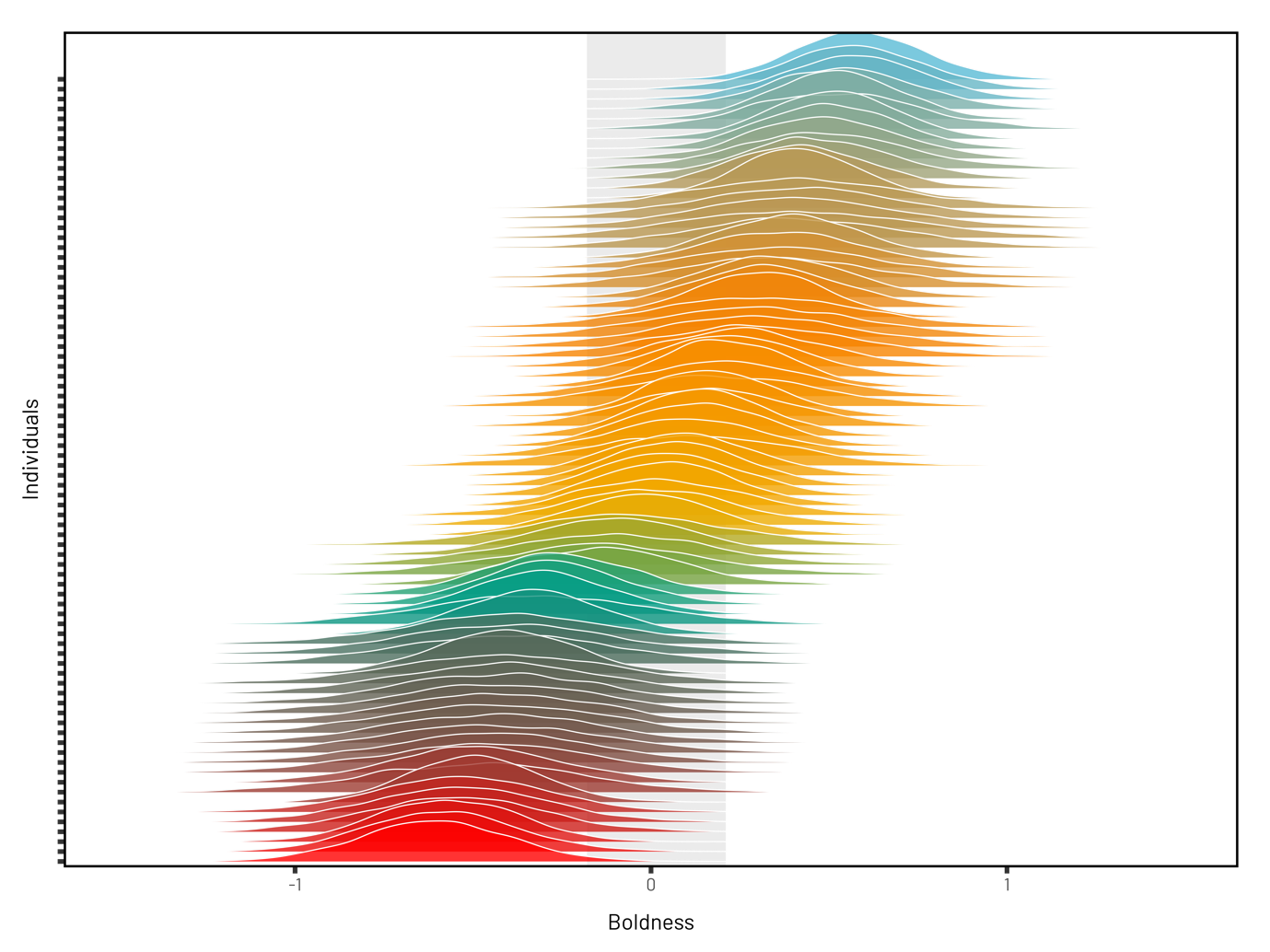


**Figure S3.1**

Posterior distributions of boldness for all sampled individuals. Each density represents a different individual. The grey shaded area represents the 89% credible intervals for the mean boldness value.

# Supplementary information S4

**Breakdown of feathers sampled in given years**

Throughout the study period there have been slight changes in what feathers were sampled in a given year and how many feathers were sampled in each year. A full breakdown of this can be found in table S4.1

**Table S4.1** The number of feathers collected in each year, split by feather type. Individuals were only sampled once within each year but could be sampled in multiple years. The number in brackets refer to how many of these individuals also have been assayed for boldness

| **Feather type** | **Year** | | | | | | | | |  |
| --- | --- | --- | --- | --- | --- | --- | --- | --- | --- | --- |
|  | 2013 | 2014 | 2015 | 2016 | 2017 | 2018 | 2019 | 2020 | 2021 | **Total** |
| P1  (Breeding) |  | 10 (4) | 11 (6) | 10 (8) | 10 (10) | 10 (10) | 11 (8) | 10 (8) | 8 (8) | **80** |
| P10  (Post-breeding) | 19 (7) | 10 (4) | 11 (6) | 10 (8) | 10 (10) |  | 11 (10) |  |  | **71** |
| Nape  (Pre-breeding) | 19 (7) | 10 (4) | 11 (6) | 10 (8) | 10 (10) | 10 (10) | 11 (8) | 10 (8) | 14 (3) | **105** |
| Total | **38** | **30** | **33** | **30** | **30** | **20** | **33** | **20** | **22** | **256** |

**
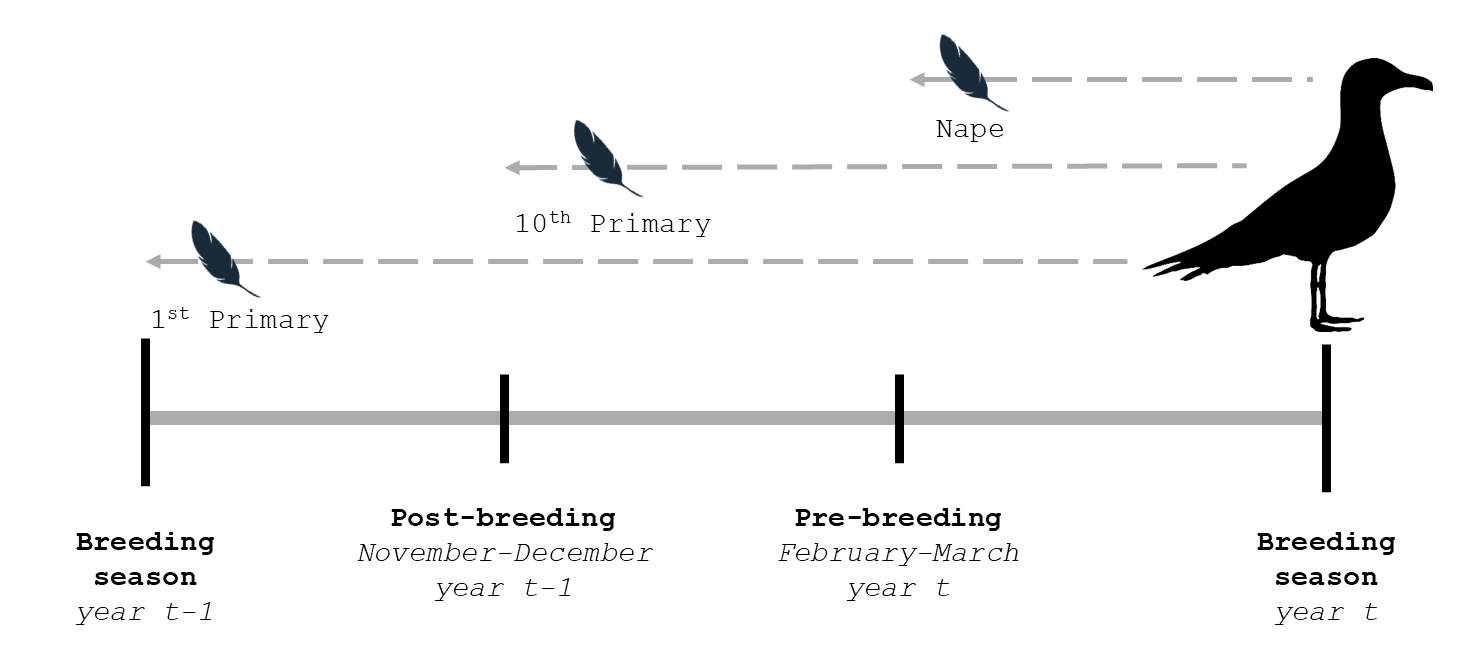
**

**Figure S4.1**

Moult timings of black-legged kittiwake *Rissa tridactyla* and how our corticosterone measures correspond to timing across the annual cycle. Moult timings are taken from (Demongin, 2016) and represent the average time these feathers are moulted.

# Supplementary information S5

**Inter- and intra assay coefficient of variation between corticosterone measures**

**Table S5.1** Calculation of inter-assay coefficient of variation (CV)

| Assay | Standard [ng/g] |
| --- | --- |
| A1 | 0.99 |
| A2 | 1.00 |
| A3 | 1.08 |
| A4 | 0.99 |
| mean | 1.02 |
| Inter-Assay CV | **4.24** |

**Table S5.2** Calculation of intra-assay coefficient of variation (CV)

| Report | Parameter | Valid N | Mean | CI -95% | CI +95% | Std.Dev. |
| --- | --- | --- | --- | --- | --- | --- |
| **Percent recovery** | % Recovery | 256 | **97.43** | 96.99 | 97.87 | **3.57** |
| **Intra-assay CV** | %Dev b/w duplicates | 256 | **1.22** | 1.10 | 1.33 | **0.93** |

# Supplementary information S6

**Raw corticosterone values**

**
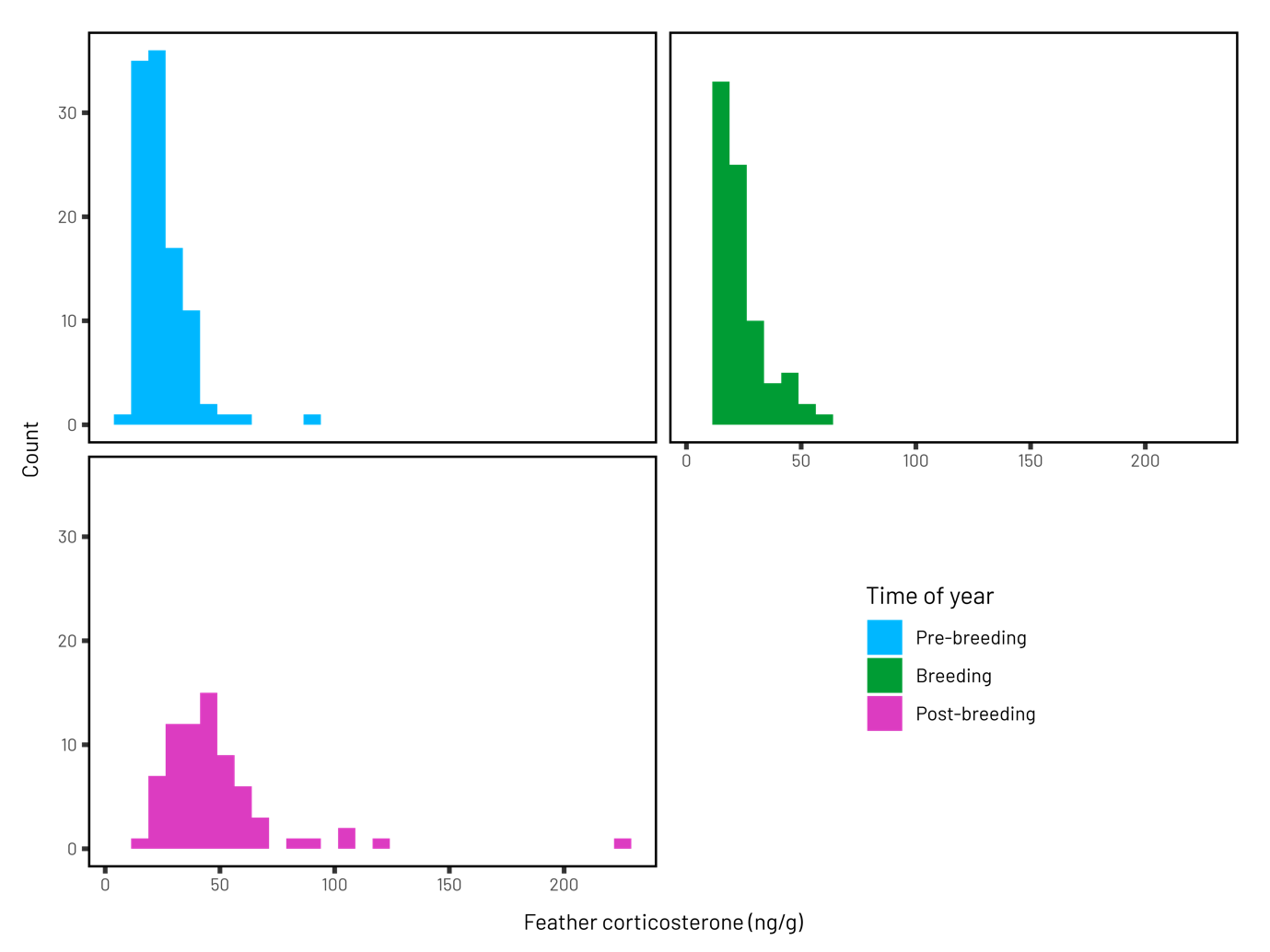
**

**Figure S6.1**

Raw feather corticosterone values in nanograms per gram for the three different feather types. (A) Pre-breeding, breeding and post-breeding periods refer to the nape, p1 and p10 feathers respectively. These correspond to (A) (nape/pre-breeding), (B) (p1/breeding) and (C) (p10/post-breeding) panels on the graph.

# Supplementary information S7

**Validation of the radioimmunoassay for kittiwake feather corticosterone**

**
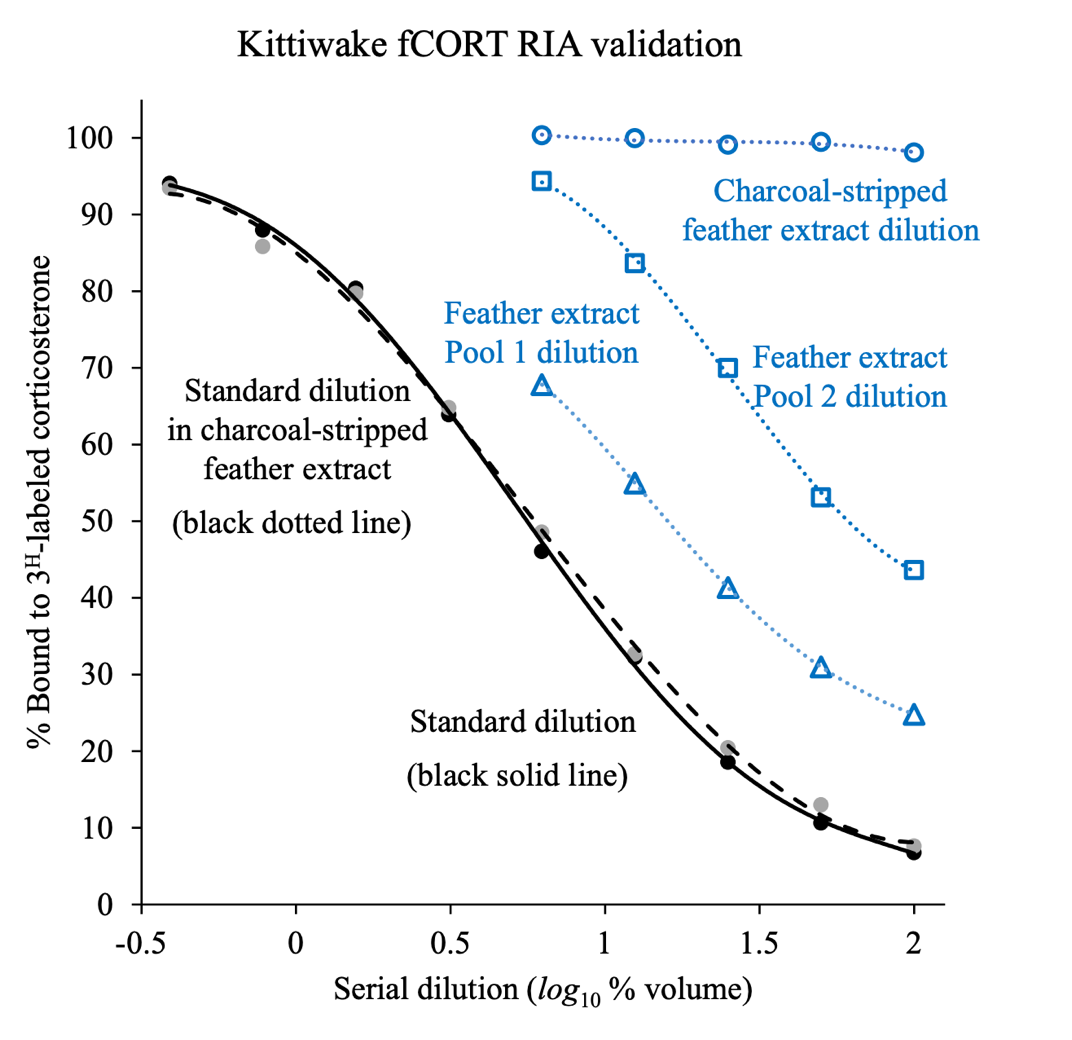
Figure S7.1**

Percent binding of the Sigma Aldrich antibody to corticosterone across serially diluted samples.

# Supplementary information S8

**Feather corticosterone as an indicator of nutritional stress**

Since the initial work by Bortolotti et al in 2008 there has been a sustained interest in whether feather corticosterone can represent periods of physiological stress in birds (Bortolotti et al., 2008; Romero and Fairhurst, 2016). While Bortolotti et al’s early work showed that feather corticosterone was positively related to stressors during moult, there are still large uncertainties surrounding how exactly corticosterone is deposited into feathers and therefore the ecological interpretations you can make from feather corticosterone (Romero and Fairhurst, 2016). Validation of feather corticosterone as a suitable measure to answer your research questions is needed to allow appropriate conclusions to be made.

Here we use feather corticosterone as a proxy of nutritional state in black-legged kittiwakes, *Rissa tridactyla*. Feather corticosterone has been shown to represent nutritional state in numerous seabird species including red-legged kittiwakes, *Rissa brevirostris*, (Will et al., 2018) and rhinoceros auklets, *Cerorhinca monocerata* (Will et al., 2019, 2015). Validation of this approach for black-legged kittiwakes has been validated through both experimental and observational studies. Firstly to validate that feather corticosterone is representative of current physiological state, baseline plasma has been shown to correlate with feather corticosterone during the same breeding season in free-living individuals (Wil et al., in prep; Figure S8.1). The same relationship is also found in birds that have had experimentally increased corticosterone through oral delivery with those treated having significantly more corticosterone than controls (Benowitz-Fredericks et al., unpublished; Figure S8.2).

**Figure S8.1**

The relationship between plasma and primary feather corticosterone concentrations in adult black-legged kittiwakes breeding on St. Lawrence Island, Alaska. Points represent raw values with the dashed line representing the model prediction. All corticosterone values are presented on the log scale.

**
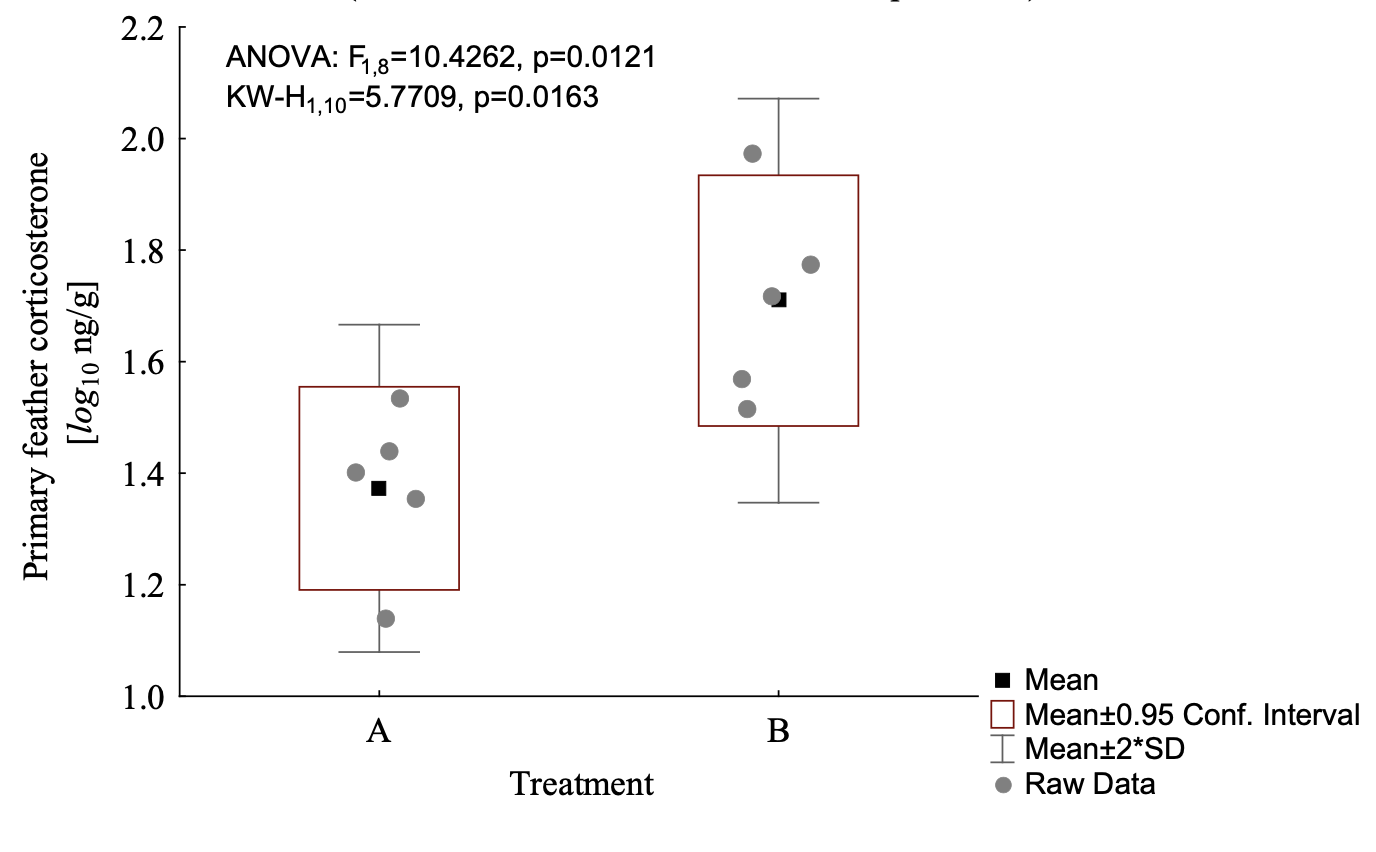
Figure S8.2**

The relationship between enogenous corticosterone treatment and primary feather corticosterone in black-legged kittiwake chicks. Treatment A represents control individuals with treatment B representing those with experimentally enhanced plasma corticosterone.

Subsequently if individuals face periods of reduced food consumption and therefore require the use of corticosterone to activate endogenous energy sources we would expect to see concurrent elevations in feather corticosterone. This is again something we see under both natural and experimental conditions. Firstly, experimentally we see that individuals fed ad libitum with capelin, *Mallotus villosus*, during moult have significantly lower feather corticosterone that unfed individuals (Kitaysky *et al*., unpublished; Figure S8.3). Secondly we find that between-year variation in feather corticosterone from feathers grown during the breeding season can be explained by pelagic fish biomass with years of high food availability correlating with lower average feather corticosterone (Will *et al*., unpublished; Figure S8.4).


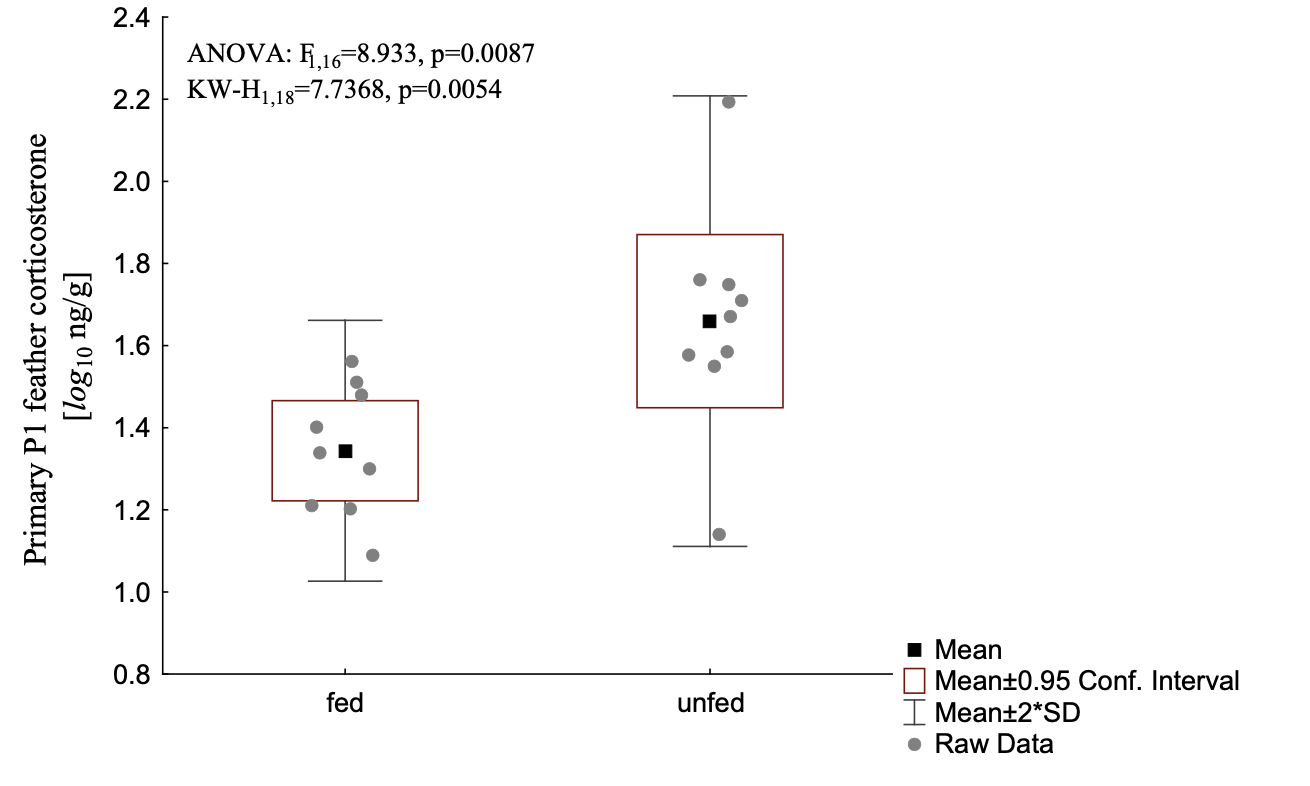
**Figure S8.3**

Feather corticosterone concentrations in primary feather fragments of food-supplemented (“fed”) and control (“unfed”) black-legged kittiwakes breeding on Middleton Island. These data come from 2015.


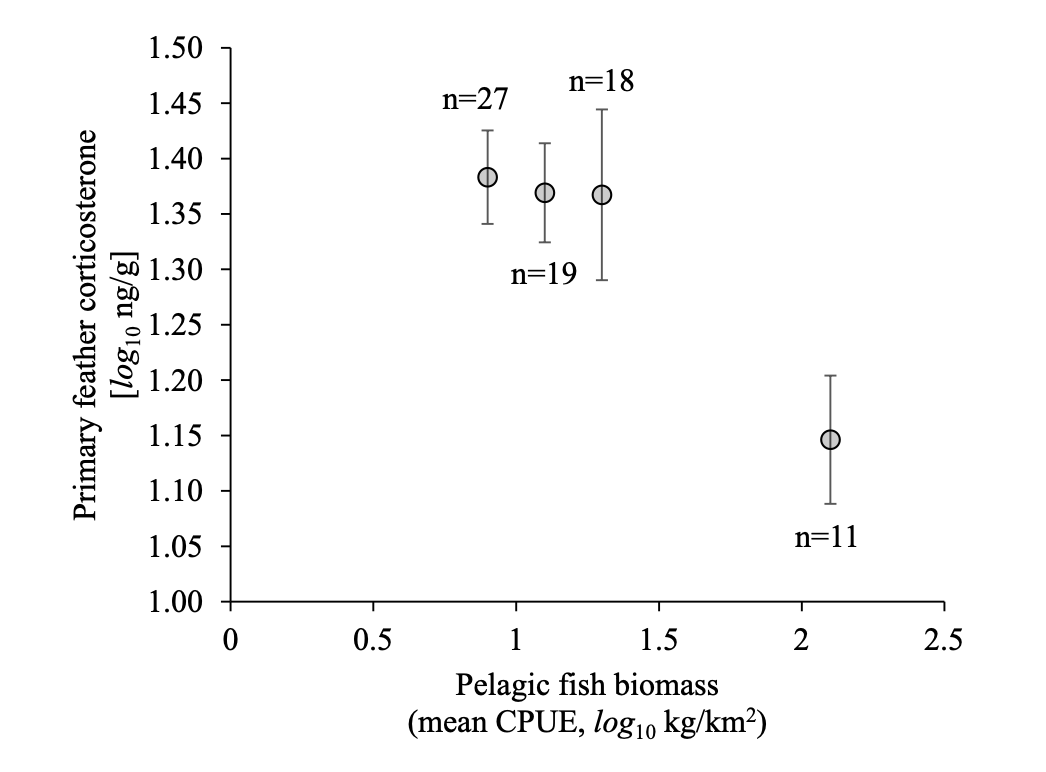


**Figure S8.4**

The relationship between primary feather corticosterone and abundance of pelagic forage fish from trawl surveys. Points represent the means with standard deviations as the error bars. Trawl surveys were conducted by NOAA.

# Supplementary information S9

**Linear vs negative exponential relationship between AWI and feather corticosterone**


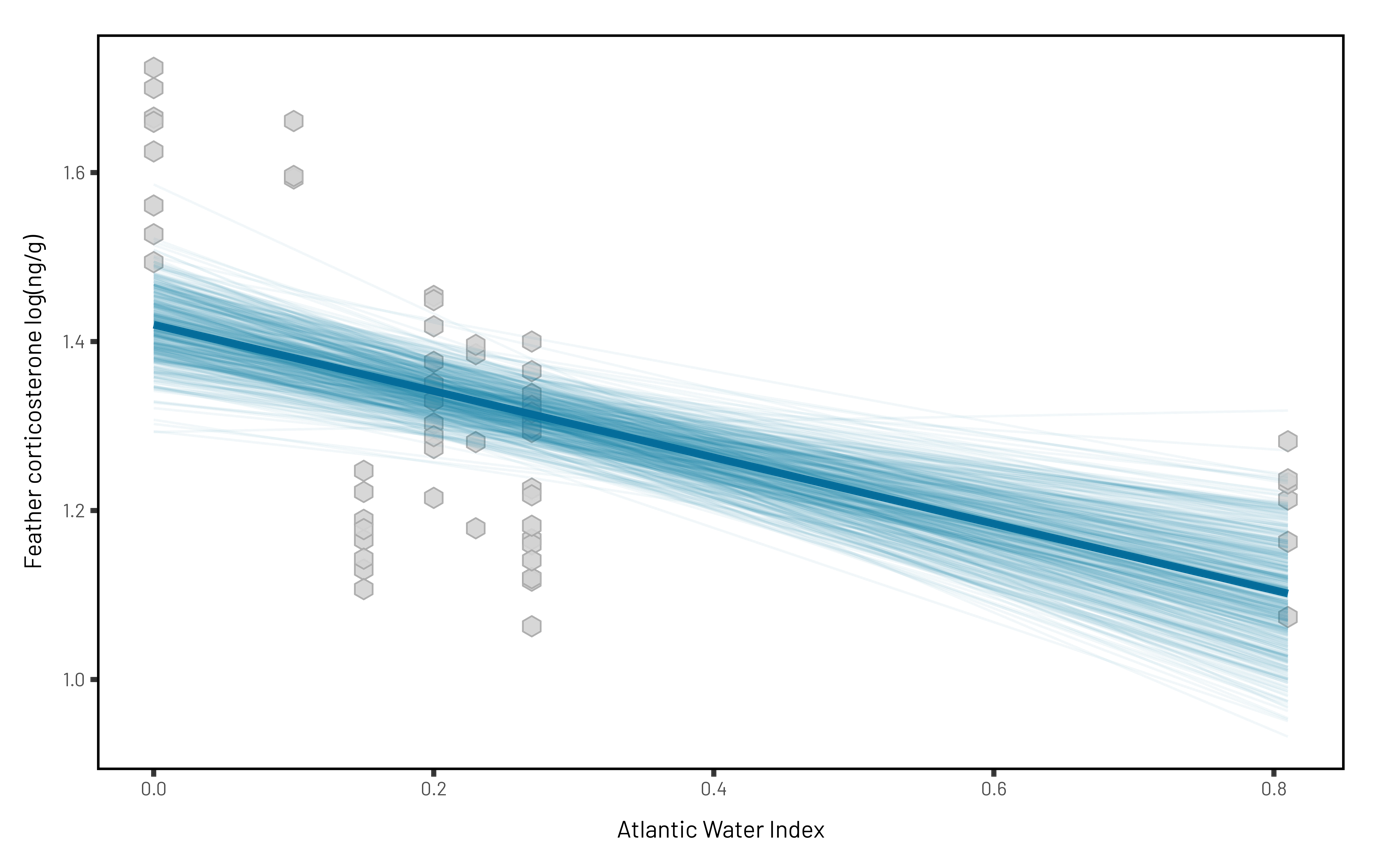


**Figure S9.1**

Linear relationship between AWI and feather corticosterone. Black line represents the model prediction with lighter and darker blues representing the 50 and 89% Credible intervals. At low AWI values, the model underpredicts feather corticosterone and so therefore a negative exponential relationship was used in the models presented in the main manuscript.

# Supplementary information S10

**Model priors**

In all models, Normal priors were used for the intercept and fixed effects. Half-cauchy priors were the most commonly used priors for the random effects and residuals. Half-Cauchy distributions are a specific Cauchy distribution that is bound to a probability density that is always non-zero and are commonly used for random effects in Bayesian modelling (Bürkner, 2017; Regan and Sheldon, 2023).

Exact prior specifications for each model can be found in Table S9.1

**Table S10.1**

Prior specifications used in the models presented in the manuscript. Numbers inside brackets refer to [mean, standard deviation] respectively.

| Model | Response | Parameter | Prior |
| --- | --- | --- | --- |
| 1 | Breeding fCORT | Intercept | Normal[1.2,1] |
|  |  | Fixed effects | Normal[0,1] |
|  |  | Random effects | Half-cauchy[0.05, 0.05] |
|  |  | Residual | Half-cauchy[0.1,0.1] |
| 2 | Pre-breeding fCORT | Intercept | Normal[1.4,1] |
|  |  | Fixed effects | Normal[0,1] |
|  |  | Random effects | Half-cauchy[0.05, 0.05] |
|  |  | Residual | Half-cauchy[0.1,0.1] |
| 3 | Post-breeding fCORT | Intercept | Normal[1.6,1] |
|  |  | Fixed effects | Normal[0,1] |
|  |  | Random effects | Half-cauchy[0.05, 0.05] |
|  |  | Residual | Half-cauchy[0.1,0.1] |
| 4 | All fCORT values | Intercept | Normal[1.5,1] |
|  |  | Fixed effects | Normal[0,1] |
|  |  | Random effects | Half-cauchy[0.05, 0.05] |
|  |  | Residual | Half-cauchy[0.1,0.1] |
| 5, 6, 7 | Chick survival to 15 days | Intercept | Normal[0,5] |
|  |  | Fixed effects | Normal[0,2] |
|  |  | Random effect | Half-cauchy[1,1] |
| 6,7,8 | Return rate | Intercept | Normal[0.75,0.25] |
|  |  | Fixed effects | Normal[0,5] |
|  |  | Random effect | Half-cauchy[1,1] |

# Supplementary information S11

**Relationship between boldness and feather corticosterone levels across the annual cycle**


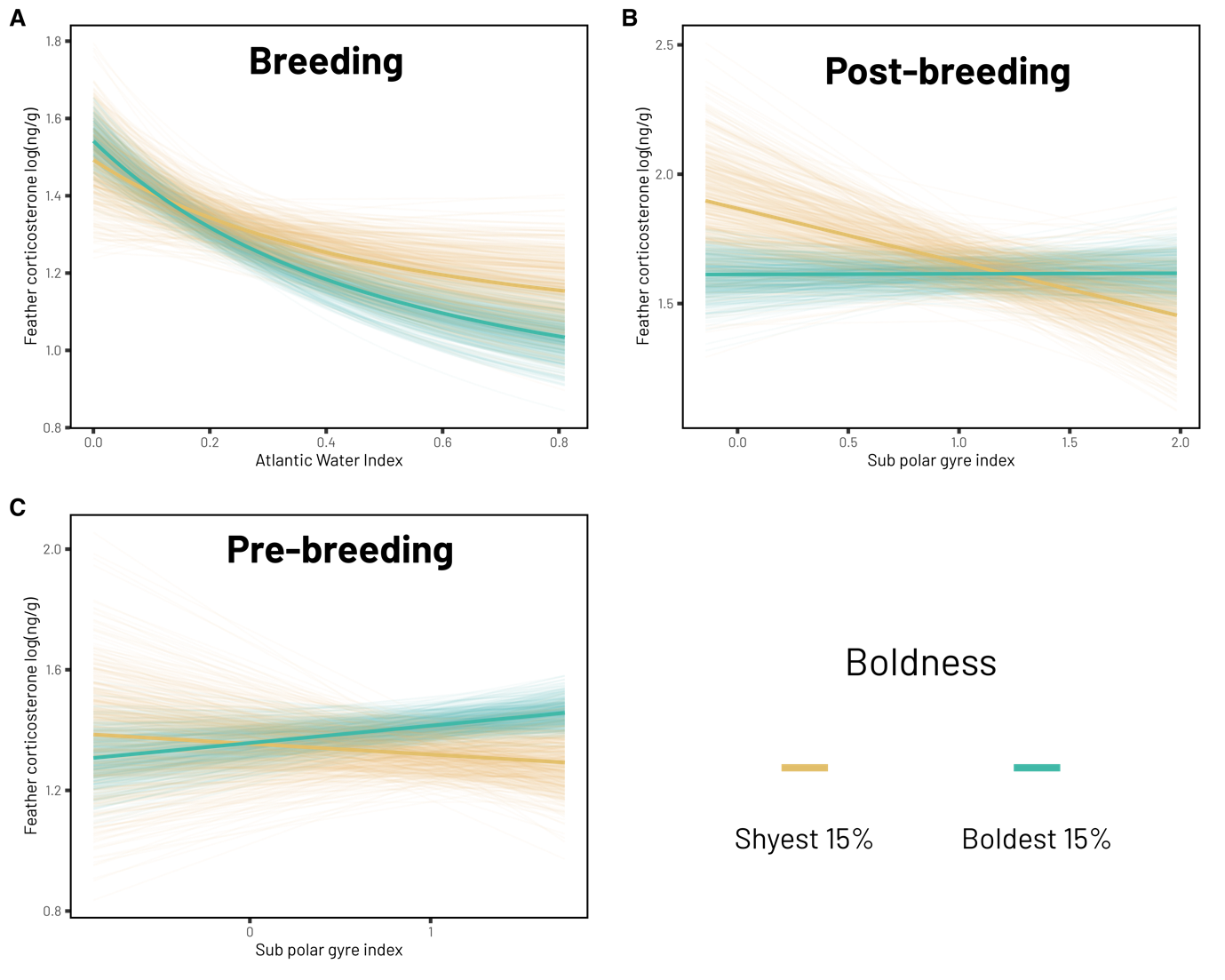


**Figure S11.1**

The relationship between environmental proxies of resource acquisition, feather corticosterone and boldness throughout the annual cycle (A: breeding season, B: post-breeding, C: pre-breeding). Thick lines represent the mean predicted relationship extracted from each model, with thinner lines representing 500 predicted draws of the most likely relationship from the posterior distributions using the add_epred_draws() function from the tidybayes packages in R (Kay & Mastny, 2023). Colours represent predictions for the shyest (“Yellow”) and boldest (“blue”) 15% of individuals respectively. All corticosterone values are represented on the log scale.

# Supplementary information S12

**Environmental determinants of corticosterone variation**


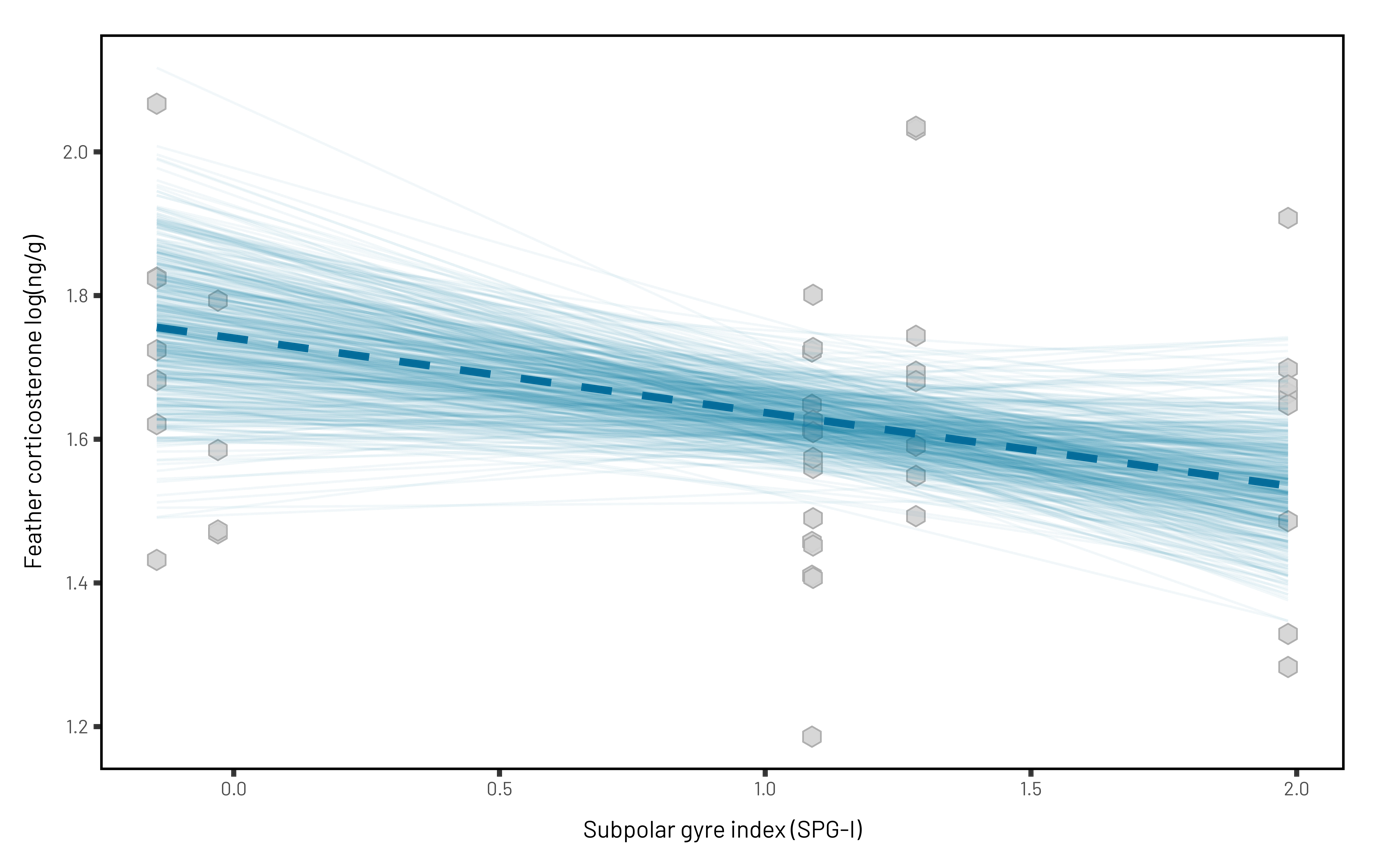


**Figure S12.1**

Relationship between the Sub-Polar Gyre Index and feather corticosterone during the post-breeding period, November-December. Grey points represent the raw data. Individual blue lines represent 500 predicted relationships between AWI and corticosterone.

**
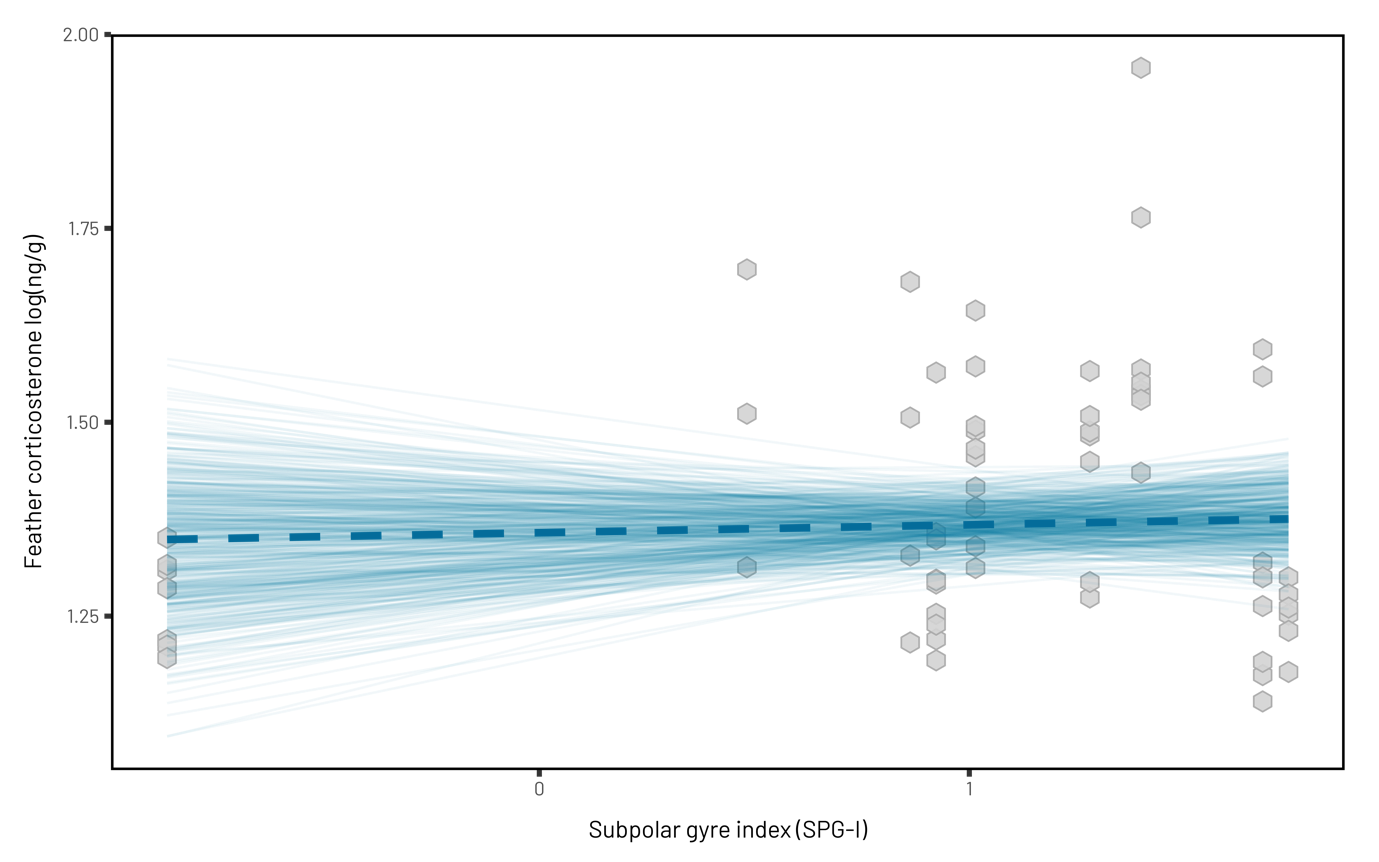
**

**Figure S12.2**

Relationship between the Sub-Polar Gyre Index and feather corticosterone during the pre-breeding period, February to March. Grey points represent the raw data. Individual blue lines represent 500 predicted relationships between AWI and.

# Supplementary information S13

**Variation in corticosterone across the annual cycle**

**
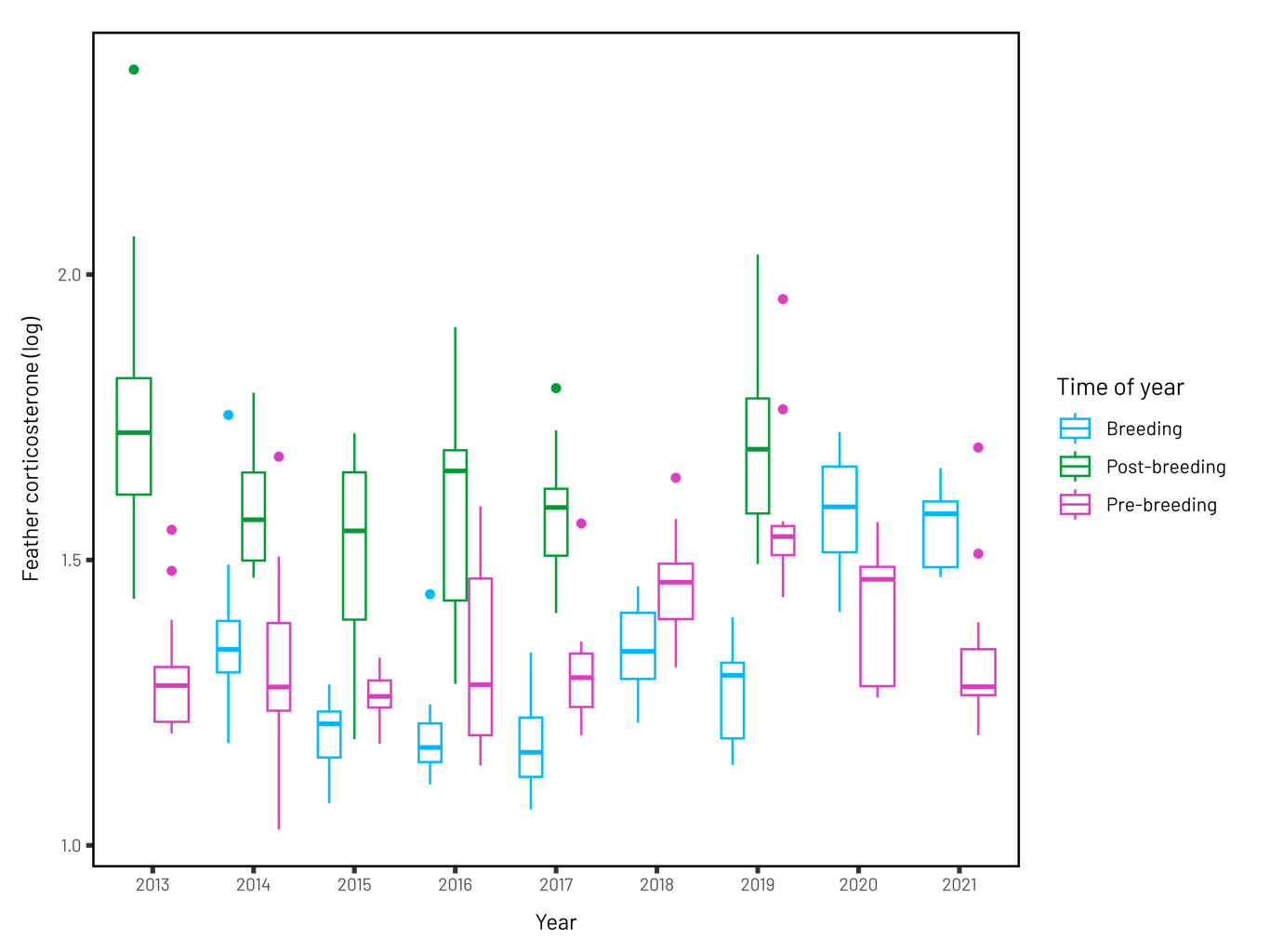
**

**Figure S13.1**

Variation in corticosterone at different times of year between 2013 and 2021. Boxplots are coloured by the time of year corticosterone was measured.


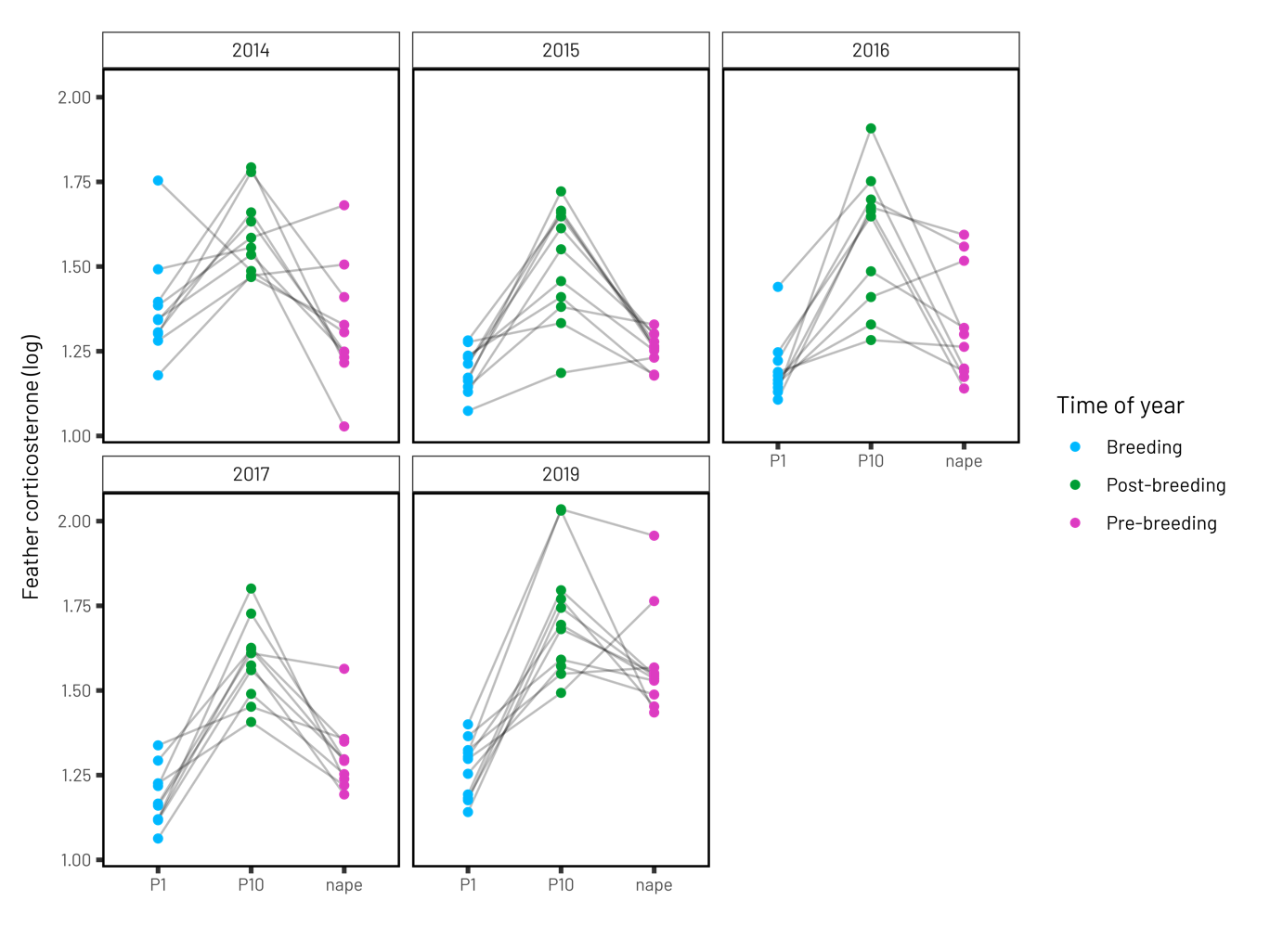
**Figure S13.2**

Variation in corticosterone within individuals across the annual cycle. Each line represents a single individual in a given year with the points coloured by when the individual corticosterone measurement was taken.

**References**

Bantock, T.M., Prys-Jones, R.P., Lee, P.L.M., 2008. New and improved molecular sexing methods for museum bird specimens. Molecular Ecology Resources 8, 519–528. https://doi.org/10.1111/j.1471-8286.2007.01999.x

Bortolotti, G.R., Marchant, T.A., Blas, J., German, T., 2008. Corticosterone in feathers is a long-term, integrated measure of avian stress physiology. Functional Ecology 22, 494–500. https://doi.org/10.1111/j.1365-2435.2008.01387.x

Coulson, J.C., 2009. Sexing Black‐legged Kittiwakes by measurement. Ringing & Migration 24, 233–239. https://doi.org/10.1080/03078698.2009.9674397

Demongin, L., 2016. Identification Guide to Birds in the Hand: The 301 Species Most Frequently Caught in Western Europe : Identification, Measurements, Geographical Variation, Moult, Sex and Age. Pelagic Publications.

Griffiths, R., Double, M.C., Orr, K., Dawson, R.J.G., 1998. A DNA test to sex most birds. Molecular Ecology 7, 1071–1075. https://doi.org/10.1046/j.1365-294x.1998.00389.x

Harris, S.M., Descamps, S., Sneddon, L.U., Bertrand, P., Chastel, O., Patrick, S.C., 2020. Personality predicts foraging site fidelity and trip repeatability in a marine predator. Journal of Animal Ecology 89, 68–79. https://doi.org/10.1111/1365-2656.13106

Kay, M., Mastny, T., 2023. tidybayes: Tidy Data and “Geoms” for Bayesian Models.

McCully, F.R., Descamps, S., Harris, S.M., Mckendrick, F., Gillies, N., Cornell, S.J., Hatchwell, B.J., Patrick, S.C., 2023. Links between personality, reproductive success and re-pairing patterns in a long-lived seabird. Ethology 129, 686–700. https://doi.org/10.1111/eth.13405

Nakagawa, S., Schielzeth, H., 2010. Repeatability for Gaussian and non-Gaussian data: a practical guide for biologists. Biological Reviews 85, 935–956. https://doi.org/10.1111/j.1469-185X.2010.00141.x

Romero, L.M., Fairhurst, G.D., 2016. Measuring corticosterone in feathers: Strengths, limitations, and suggestions for the future. Comparative Biochemistry and Physiology Part A: Molecular & Integrative Physiology, Ecophysiology methods: refining the old, validating the new and developing for the future 202, 112–122. https://doi.org/10.1016/j.cbpa.2016.05.002

Will, A., Watanuki, Y., Kikuchi, D.M., Sato, N., Ito, M., Callahan, M., Wynne-Edwards, K., Hatch, S., Elliott, K., Slater, L., Takahashi, A., Kitaysky, A., 2015. Feather corticosterone reveals stress associated with dietary changes in a breeding seabird. Ecol. Evol. 5, 4221–4232. https://doi.org/10.1002/ece3.1694

Will, A., Wynne-Edwards, K., Zhou, R., Kitaysky, A., 2019. Of 11 candidate steroids, corticosterone concentration standardized for mass is the most reliable steroid biomarker of nutritional stress across different feather types. Ecology and Evolution 9, 11930–11943. https://doi.org/10.1002/ece3.5701

Will, A.P., Kitaiskaia, E.V., Kitaysky, A.S., 2018. Red-legged kittiwake feathers link food availability to environmental changes in the Bering Sea. Marine Ecology Progress Series 593, 261–274. https://doi.org/10.3354/meps12509
